# ZNF354C is a transcriptional repressor that inhibits endothelial angiogenic sprouting

**DOI:** 10.1038/s41598-020-76193-0

**Published:** 2020-11-05

**Authors:** James A. Oo, Barnabas Irmer, Stefan Günther, Timothy Warwick, Katalin Pálfi, Judit Izquierdo Ponce, Tom Teichmann, Beatrice Pflüger-Müller, Ralf Gilsbach, Ralf P. Brandes, Matthias S. Leisegang

**Affiliations:** 1 Institute for Cardiovascular Physiology, Goethe University, Theodor-Stern-Kai 7, 60590 Frankfurt am Main, Germany; 2 Max Planck Institute for Heart and Lung Research, Bad Nauheim, Germany; 3 German Center of Cardiovascular Research (DZHK), Partner site RheinMain, Frankfurt, Germany

**Keywords:** Angiogenesis, Gene regulation, Transcriptional regulatory elements

## Abstract

Zinc finger proteins (ZNF) are a large group of transcription factors with diverse functions. We recently discovered that endothelial cells harbour a specific mechanism to limit the action of ZNF354C, whose function in endothelial cells is unknown. Given that ZNF354C has so far only been studied in bone and tumour, its function was determined in endothelial cells. ZNF354C is expressed in vascular cells and localises to the nucleus and cytoplasm. Overexpression of ZNF354C in human endothelial cells results in a marked inhibition of endothelial sprouting. RNA-sequencing of human microvascular endothelial cells with and without overexpression of ZNF354C revealed that the protein is a potent transcriptional repressor. ZNF354C contains an active KRAB domain which mediates this suppression as shown by mutagenesis analysis. ZNF354C interacts with dsDNA, TRIM28 and histones, as observed by proximity ligation and immunoprecipitation. Moreover, chromatin immunoprecipitation revealed that the ZNF binds to specific endothelial-relevant target-gene promoters. ZNF354C suppresses these genes as shown by CRISPR/Cas knockout and RNAi. Inhibition of endothelial sprouting by ZNF354C is dependent on the amino acids DV and MLE of the KRAB domain. These results demonstrate that ZNF354C is a repressive transcription factor which acts through a KRAB domain to inhibit endothelial angiogenic sprouting.

## Introduction

The vascular system is controlled by numerous signaling pathways and growth factors which all contribute to the regulation of gene expression. Transcription factors (TF) are one of the largest protein groups that exist in humans. TF directly control gene expression; a process which is relevant for most cellular functions and for disease development^1,2^. TFs with zinc-coordinating domains represent the predominant superclass of TFs in humans^1,3,4^. Zinc finger proteins (ZNFs) are important for differentiation, proliferation, the maintenance of tissue integrity and cellular homeostasis. These fundamental functions imply their involvement in tumour formation, neurodegeneration, cardiovascular diseases and many other pathophysiological processes^4^.

In the cardiovascular system, the well-studied TFs, GATA4 and GATA6, both contribute to cardiac morphogenesis and are associated with congenital heart disease^5^. Examples of C2H2-type ZNFs that regulate angiogenesis are Vascular Endothelial Zinc Finger 1 (Vezf1) and ZNF350. Vezf1 is required for proliferation, migration and network formation of murine endothelial cells^6^. ZNF350, a Krüppel-associated box containing ZNF (KRAB-ZNF), is a potent transcriptional inhibitor of Angiopoietin-1 acting with BRCA1^7^. Krüppel-like transcription factors (KLFs) are central for endothelial function and homeostasis^8,9^: KLF4, together with KLF2, organises the endothelial response to shear stress including the expression of nitric oxide synthase and thrombomodulin^9,10^.

ZNF354C is a C2H2-type ZNF expressed in foetal brain and kidney, which has been associated with bone development and progression of oesophageal squamous cell carcinoma^11–13^. We previously reported that ZNF354C is active in human umbilical vein endothelial cells: it inhibits transcription of sphingosine-1-phosphate receptor-1 (S1PR1) and endothelial cells specifically express the long non-coding RNA “long intergenic noncoding RNA antisense to S1PR1” (LISPR1) to prevent the interaction of ZNF354C with the endothelial S1PR1 promoter^14^. Despite this observation, ZNF354C is poorly characterised in the cardiovascular system. As, however, ZNF354C binds the short DNA consensus motif 5′-NNCCAC-3′ in humans (5′-CCACA-3′ in rats)^11,12,15^, which is contained in hundreds of gene promoters, a very important function of ZNF354C can be inferred. Therefore, we hypothesise that ZNF354C is an important protein in the cardiovascular system contributing to vascular transcriptional regulation.

## Results

### ZNF354C is an important inhibitor of cellular endothelial angiogenic sprouting in culture

**Figure 1:**
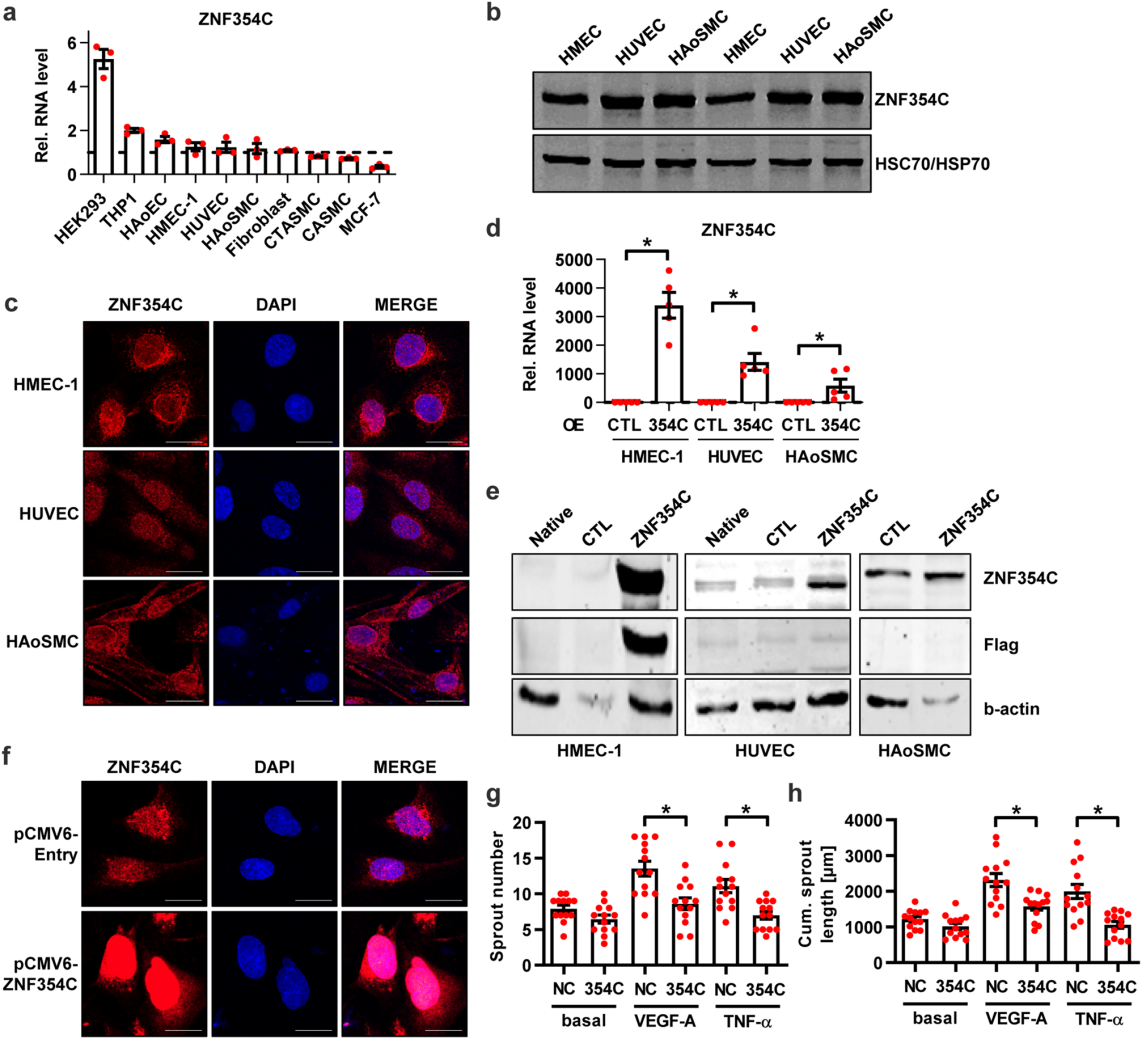
Zinc Finger Protein 354C (ZNF354C) overexpression leads to inhibition of sprouting in HMEC-1. (**a**) RNA expression level as measured with RT-qPCR of ZNF354C in different cell types. Data is normalised to HUVEC batch 1 (set as 1) and to b-actin. n = 3. Data are mean ± SEM. (**b**) Representative western blot of HUVEC, HMEC-1 and HAoSMC. ZNF354C antibody was used. HSC70/HSP70 antibodies served as loading control. (**c**) Immunofluorescence with an antibody against ZNF354C in HMEC-1, HUVEC and HAoSMC. Nuclei were stained with DAPI. Scale bar indicates 20 µm. (**d**) RT-qPCR of ZNF354C after overexpression (OE) of pCMV6-ZNF354C (354C) in HMEC-1, HUVEC and HAoSMC. PCMV6-entry was used as negative control (CTL). Data is normalized to b-actin. n = 5. Data are mean ± SEM. Mann–Whitney t-test; *P < 0.05. (**e**) Representative western blot of untreated (native) HMEC-1, HUVEC and HAoSMC or after overexpression of pCMV6-ZNF354C (ZNF354C) or pCMV6-entry (CTL). ZNF354C, Flag or b-actin antibodies were used. (**f**) Immunofluorescence with an antibody against ZNF354C in HMEC-1 after overexpression of pCMV6-ZNF354C or pCMV6-entry. Nuclei were stained with DAPI. Scale bar indicates 20 µm. (**g**, **h**) Spheroid outgrowth assay quantification of sprout number (**g**) and cumulative sprout length (**h**) after overexpression of pCMV6-ZNF354C (354C) or pCMV6-entry (NC). Cells were studied under basal conditions or after treatment with VEGF-A or TNF-α. Scale bar indicates 50 µm. n = 13. One-Way ANOVA with Bonferroni post-hoc test. *P < 0.05.

We previously demonstrated that ZNF354C inhibits transcription of S1PR1 in HUVECs and therefore limits S1P signaling. This prompted us to characterise the physiological and molecular role of ZNF354C in the vascular system as almost nothing is known about this ZNF354C in the cardiovascular context. RNA and protein expression and Encode Cap Analysis of Gene Expression (CAGE) promoter analysis revealed that ZNF354C is expressed in all tissues tested but, most importantly, it is strongly expressed in vascular cells such as human umbilical vein endothelial cells (HUVECs), human microvascular endothelial cells-1 (HMEC-1) and human aortic smooth muscle cells (HAoSMCs) (Fig. 1a,b, Sup. Fig. 1a,b). To evaluate the intracellular localisation of ZNF354C in HUVECs, HMEC-1 and HAoSMCs, we performed immunofluorescence with antibodies targeting ZNF354C. This demonstrated that ZNF354C localises to both the nucleus and the cytoplasm and additionally accumulates at the nuclear membrane (Fig. 1c).

To identify the physiological role of ZNF354C, gain-of-function approaches were used on different vascular cell types. To increase ZNF354C protein levels, overexpression of ZNF354C was performed in HMEC-1, HUVECs and HAoSMCs. 24 h after transfection with a C-terminally DDK-Myc-tagged ZNF354C plasmid, every cell type displayed an increased ZNF354C mRNA expression (Fig. 1d) but a marked increase in protein abundance could only be achieved in HMEC-1 (Fig. 1e). Immunofluorescence in HMEC-1 with antibodies against ZNF354C after overexpression reflected the strong protein expression (Fig. 1f). Next, we investigated whether ZNF354C impacts endothelial cell function. Overexpression approaches in HMEC-1 revealed that ZNF354C inhibited angiogenic sprouting in response to vascular endothelial growth factor A (VEGF-A) and tumor necrosis factor α (TNF-α) in the spheroid outgrowth assay (Fig. 1g,h).

### ZNF354C overexpression leads to the transcriptional repression of many genes

**Figure 2:**
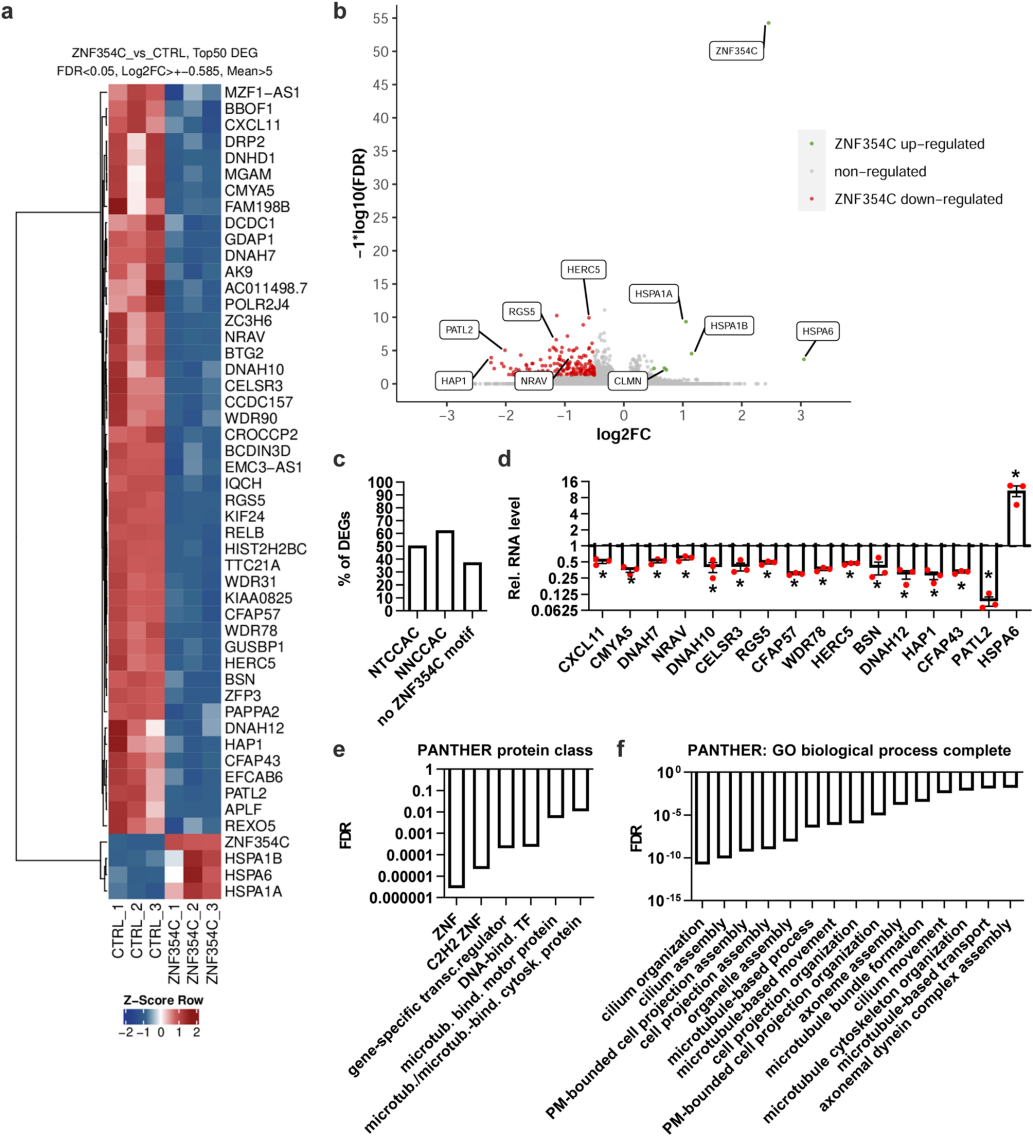
ZNF354C is a putative transcriptional repressor in HMEC-1. (**a**) Heat-map of RNA-Seq after overexpression of pCMV6-ZNF354C (ZNF354C) or pCMV6-Entry (CTRL). 3 replicates are shown. Top50 differentially expressed genes (DEG) were selected according to their p-value stringency (False discovery rate (FDR) < 0.05), logarithmic fold change (Log2FC >  ± 0.585) and number of mean reads in both groups (> 5). Z-Score represents up- (red, positive value) or downregulation (blue, negative values) of genes after overexpression of ZNF354C. (**b**) Volcano plot of RNA-Seq after overexpression of pCMV6-ZNF354C (ZNF354C) or pCMV6-Entry (CTRL). Conditions are adjusted p-value < 0.05 (FDR); Colored points indicate genes significantly differentially expressed (log2FC >  ± 0.5). Red, ZNF354C downregulated genes; green, ZNF354C upregulated genes; grey, non-regulated genes (log2FC <  ± 0.5). (**c**) Percentage of DEGs harboring the NTCCAC, NNCCAC or none of these motifs in their promoter (0-500 bp upstream of the transcriptional start site). (**d**) RT-qPCR of the transcripts indicated after overexpression of pCMV6-ZNF354C in HMEC-1. PCMV6-entry was used as negative control (CTL) and set as 1. Data is normalised to b-actin. n = 3. Data are mean ± SEM. Paired t-test; *P < 0.05. (**e**, **f**) Gene ontology (GO) enrichment analysis with PANTHER for protein classes (**e**) or biological process complete (**f**) terms. Only downregulated DEG (logFC > 0.3) were used. Fisher’s exact test; FDR, false discovery rate.

To identify the impact of ZNF354C on gene expression, mRNA-sequencing was performed in HMEC-1 (Fig. 2a, Sup. Table 1). As compared to control transfected cells, ZNF354C overexpression mainly decreased gene expression (Fig. 2b). In detail, 167 genes were significantly (padj.) downregulated with a log2 fold change (logFC) of more than 0.5, whereas only 7 genes were upregulated (Sup. Fig. 2a,b). A screen for the presence of the ZNF354C motifs in the promoters of the differentially regulated genes (DEGs) revealed that more than 60% of these contain the sequence 5′-NNCCAC-3′ within the 0–500 bp upstream of their transcriptional target site (Fig. 2c). RT-qPCR after overexpression of ZNF354C confirmed the RNA-seq results (Fig. 2d). Gene set enrichment analysis of the downregulated genes (logFC > 0.3) revealed “ZNFs”, primary cilium and microtubule assembly/ organisation processes to be enriched in the ZNF354C regulated genes (Fig. 2e,f). This suggests that ZNF354C acts as a direct transcriptional repressor for the majority of the DEGs in endothelial cells.

### Specific mutations of the KRAB-A box abolish the repressive effects of ZNF354C on endothelial cells

**Figure 3:**
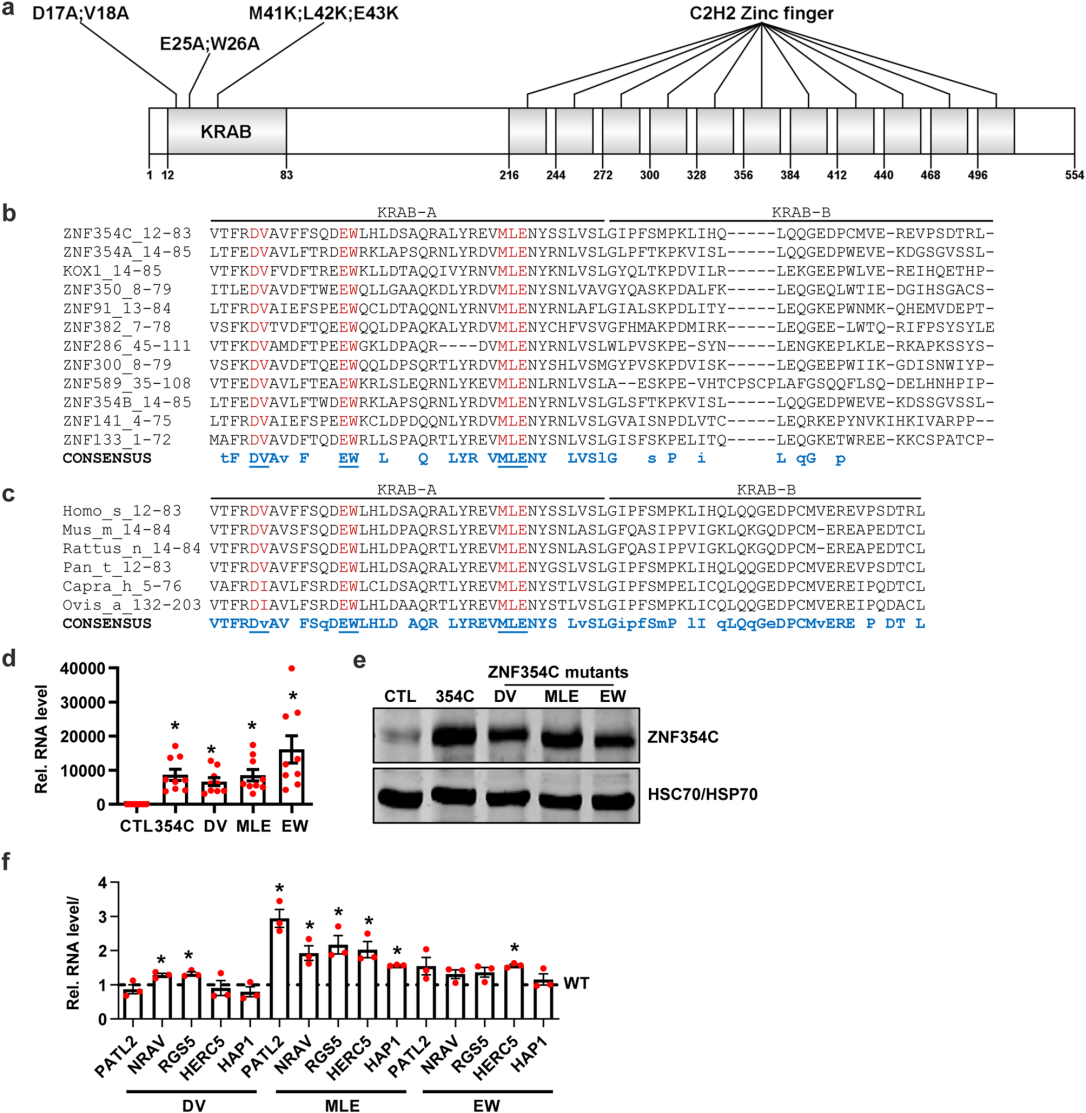
The conserved amino acid sequence MLE within the KRAB domain of ZNF354C mediates transcriptional repression. (**a**) Schematic of the ZNF354C protein architecture with its KRAB domain and 11 C2H2 Zinc fingers. Amino acids within the KRAB domain as targets for site-directed mutagenesis are indicated. Numbers below the protein indicate the amino acid position. (**b**) Alignment of KRAB sequences of various ZNF proteins with ZNF354C as a reference. The numbers within the protein names indicate the amino acid positions within the respective protein. The underlined (blue) and red amino acids were subjected to site-directed mutagenesis. (**c**) Alignment of the human ZNF354C KRAB sequence with other mammalian ZNF354C homologues. The numbers within the protein names indicate the amino acid positions within the respective protein. The underlined (blue) and red amino acids were subjected to site-directed mutagenesis. (**d**, **e**) RT-qPCR (**d**) and western blot (**e**) of ZNF354C after overexpression of pCMV6-ZNF354C (354C) or the individual mutants DV (DV → AA), MLE (MLE → KKK) or EW (EW → AA). pCMV6-Entry served as negative control (CTL). Data in d is normalised to b-actin. n = 9. Data are mean ± SEM. Paired t-test; *P < 0.05. In e, antibodies against ZNF354C and HSC70/HSP70 are shown. (f) RT-qPCR of RGS5, PATL2, HAP1, NRAV and HERC5 after overexpression of pCMV6-ZNF354C (WT, dotted line, set as 1) or the individual mutants DV (DV → AA), MLE (MLE → KKK) or EW (EW → AA). Data is normalised to b-actin. n = 3. Data are mean ± SEM. Paired t-test; *P < 0.05.

The KRAB domain has been shown to be critical for transcriptional repression in KRAB-ZNFs and mutations in the KRAB-A box disrupt transcriptional repression^16–18^ (Fig. 3a). The critical amino acids of the KRAB-A box are also present in ZNF354C (Fig. 3b,c). To determine whether these amino acids of the A-Box are required for the function of ZNF354C, three pCMV6-ZNF354C mutant constructs were generated (DV → AA, EW → AA, and MLE → KKK) by site-directed mutagenesis (Fig. 3a). Overexpression of the MLE → KKK mutant in HMEC-1 resulted in higher target gene expression than the WT plasmid (dotted line). The DV → AA as well as the EW → AA only partially increased target gene expression (Fig. 3d–f). This suggests that the MLE sequence mediates target gene transcriptional repression in ZNF354C.

### ZNF354C co-precipitates on specific target gene promoters and interacts with dsDNA, TRIM28 and Histone 3

**Figure 4:**
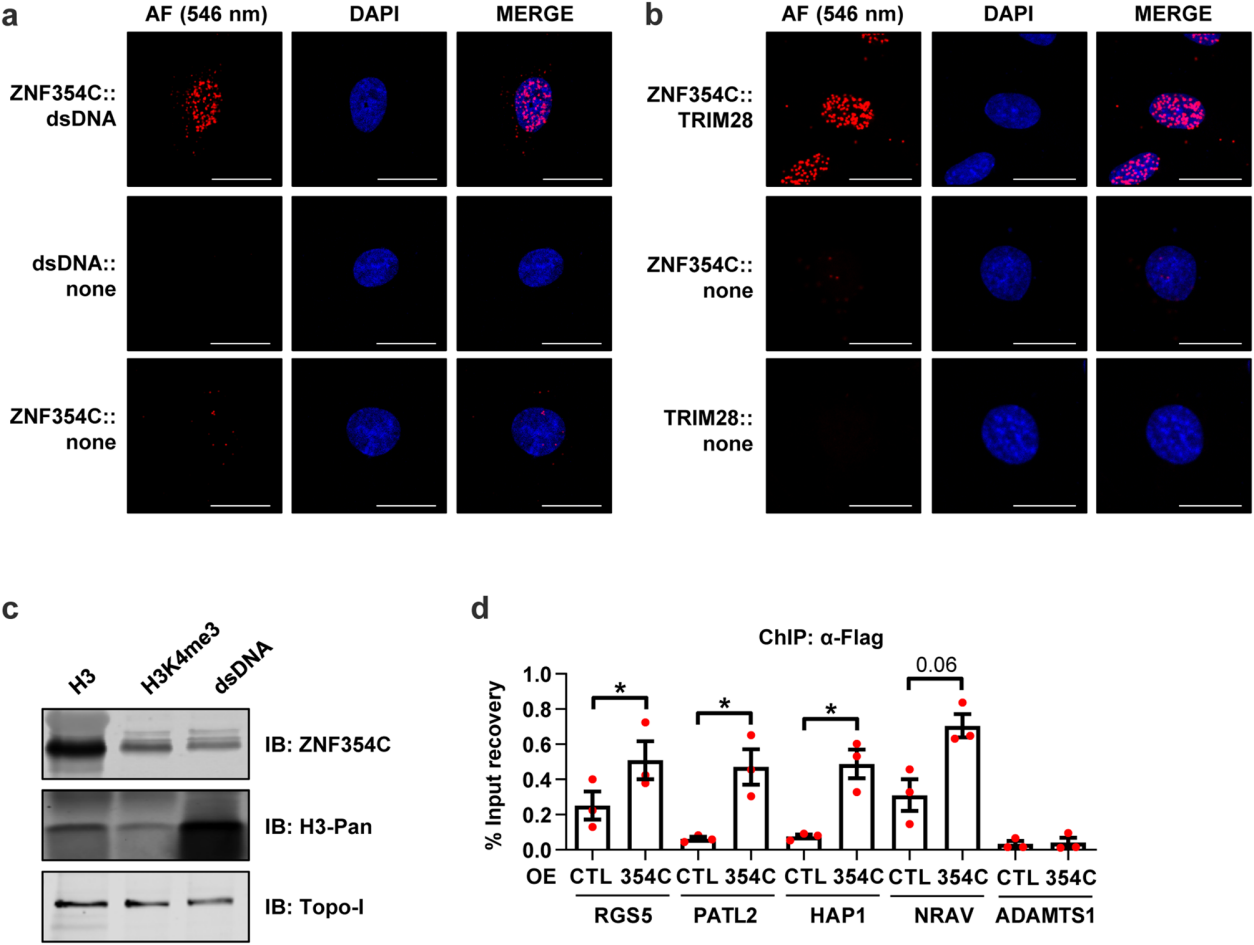
ZNF354C interacts with dsDNA, TRIM28 and histones and binds to specific target gene promoters. (**a**, **b**) Proximity ligation assay (PLA) of HMEC-1 for ZNF354C with dsDNA (**a**) or TRIM28 (**b**). ZNF354C, dsDNA or TRIM28 alone served as negative control. Red dots indicate polymerase-amplified interaction signals. Scale bar indicates 20 µm. (**c**) Immunoprecipitation with antibodies against histone 3 (H3), histone 3 lysine 4 trimethylation (H3K4me3) or dsDNA followed by western analysis. Immunoblot was performed with antibodies against ZNF354C, H3-pan and Topoisomerase I (TopoI). (**d**) Chromatin immunoprecipitation (ChIP) of HMEC-1 with antibodies against Flag after overexpression (OE) of pCMV6-ZNF354C (354C). PCMV6-entry was used as negative control (CTL). Promoter regions close to the transcriptional start site of the selected target genes RGS5, PATL2, HAP1 and NRAV was investigated by qPCR. ADAMTS1 served as negative control. n = 3. Data are mean ± SEM. Paired t-test; *P < 0.05.

KRAB-ZNFs bind to DNA with the zinc finger domains and many interact with Tripartite Motif Containing 28 (TRIM28, also known as KRAB-associated protein 1 or TIF1B) via their KRAB domain to mediate transcriptional repression^17,19^. Using proximity ligation assays, an interaction of ZNF354C with dsDNA and TRIM28 was also observed in endothelial cells (Fig. 4a,b). Immunoprecipitation with antibodies targeting dsDNA, Histone 3 or the Histone 3 modification lysine 4 trimethylation (H3K4me3) confirmed these findings and also demonstrated that ZNF354C interacts with chromatin (Fig. 4c). Overexpression of ZNF354C in HMEC-1 followed by chromatin immunoprecipitation revealed that ZNF354C indeed binds promoter sequences close to the transcriptional start site of the selected target genes Regulator Of G Protein Signaling 5 (RGS5), Protein Associated With Topoisomerase II Homolog 2 (PATL2), Huntingtin associated protein 1 (HAP1) and the long non-coding RNA Negative Regulator Of Antiviral Response (NRAV) (Fig. 4d). Importantly, RGS5 is relevant in the cardiovascular system as it promotes tumour growth^20^. No binding was observed for the negative control ADAM Metallopeptidase with Thrombospondin Type 1 Motif 1 (ADAMTS1) (Fig. 4d). Thus, ZNF354C regulates transcriptional repression through direct DNA binding.

### LentiCRISPRv2-mediated knockout or siRNA-mediated depletion of ZNF354C increases target gene expression

**Figure 5:**
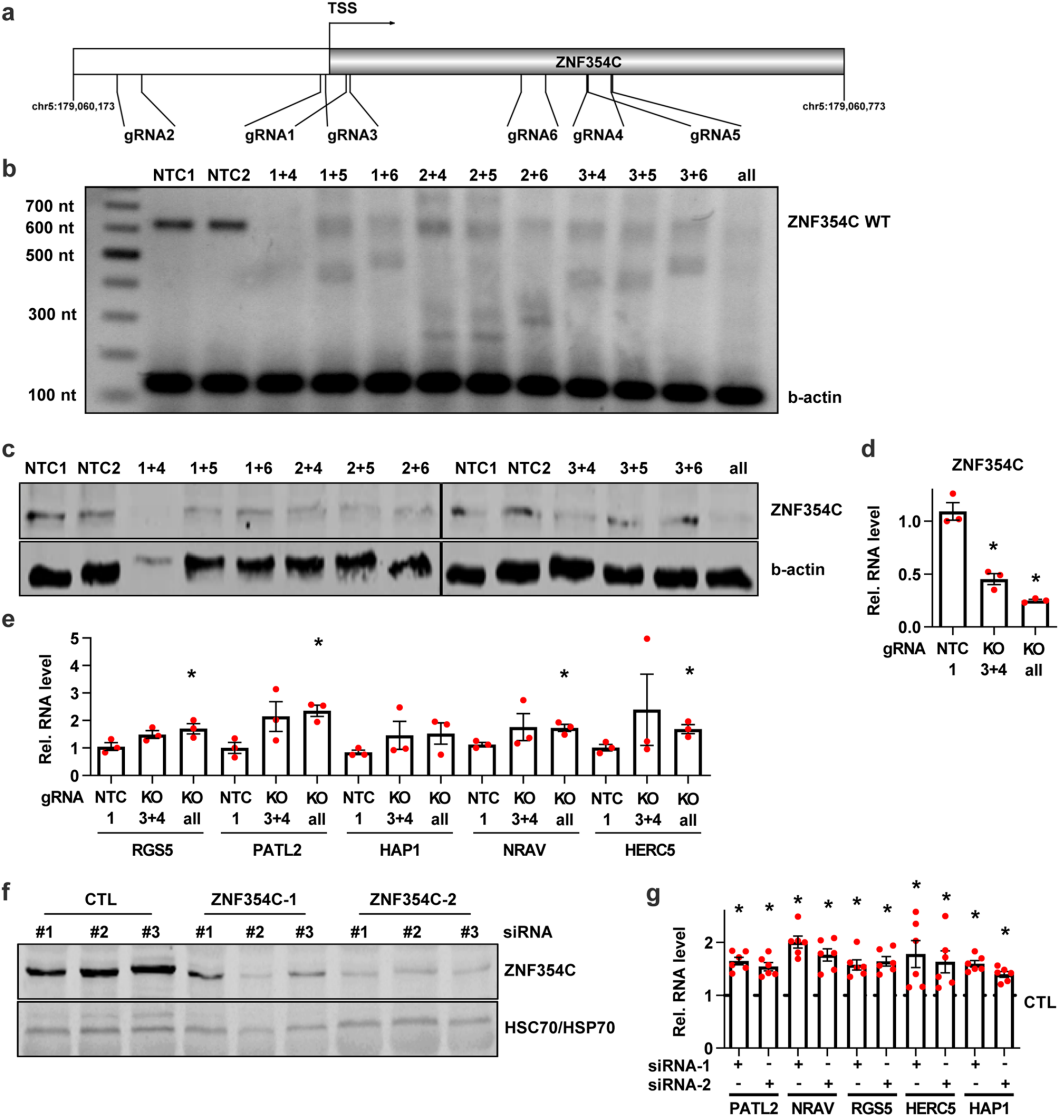
Knockout or knockdown of ZNF354C by lentiviral CRISPR/Cas9 or siRNAs increases target gene expression. (**a**) Scheme of the ZNF354C locus and the guide RNAs (gRNA) used for LentiCRISPRv2. (**b**) PCR of genomic DNA of HMEC-1 of the individual gRNA combinations used to destroy the transcriptional start site of ZNF354C. Primers targeting ZNF354C or b-actin (as loading control) were used. NTC1 and NTC2 are non-targeting negative controls showing the expected ZNF354C wild-type fragment size of 628 nt. (**c**) Western blot of the individual gRNA combinations used for LentiCRISPRv2 of ZNF354C. Antibodies against ZNF354C and b-actin (loading control) were used. NTC1 and NTC2 served as negative controls indicating the expected ZNF354C band. (**d**, **e**) RT-qPCR of ZNF354C (**d**) and RGS5, PATL2, HAP1, NRAV and HERC5 (**e**) in the knockout (KO) conditions gRNA 3 + 4 (3 + 4) and all gRNAs (all). NTC1 served as negative control. Data is normalised to b-actin. n = 3. Data are mean ± SEM. Paired t-test; *P < 0.05. (**f**) Western blot after transfection of HMEC-1 with siRNAs targeting ZNF354C (− 1, − 2) for 72 h. Scrambled siRNA (CTL) served as negative control. ZNF354C and HSC70/HSP70 antibodies were used. (**g**) RT-qPCR of RGS5, PATL2, HAP1, NRAV and HERC5 after siRNA knockdown of ZNF354C (siRNA-1, siRNA-2) in HMEC-1 for 72 h. Scrambled siRNA (CTL) served as negative control and is set as 1. Data is normalised to b-actin. n = 6. Data are mean ± SEM. Paired t-test; *P < 0.05.

In order to identify the physiological target genes of ZNF354C in endothelial cells, loss of function experiments were performed. Genes were either knocked out by CRISPR/Cas9 or down-regulated with siRNAs. LentiCRISPRv2 with 6 different guide RNAs (gRNAs) targeting genomic sequences 5′ and 3′ of the transcriptional start site of ZNF354C were used to produce a knockout of ZNF354C in HMEC-1 (Fig. 5a). After puromycin selection, genotyping revealed a positive outcome for most of the gRNA combinations (Fig. 5b); however, only a combination of all gRNAs strongly reduced ZNF354C protein and mRNA expression (Fig. 5c,d). HMEC-1 subjected to CRISPR-mediated knockout had an approximately twofold higher expression of the selected target genes, among them RGS5 (Fig. 5e).

To substantiate these results, knockdown of ZNF354C was performed with two different siRNAs in HMEC-1, HUVECs and HAoSMCs. Surprisingly, although ZNF354C mRNA expression levels strongly decreased, no obvious effect was detected on protein level after 48 h (Sup. Fig. 3a,b). Application of the protein synthesis inhibitor cycloheximide revealed that ZNF354C is a highly stable protein with a low turnover rate whose nuclear expression only starts to decrease after 48 h of translational inhibition (Sup. Fig. 3c,d). Nonetheless, knockdown of ZNF354C in HMEC-1 for 72 h revealed decreased protein levels (Fig. 5f) and demonstrated increased expression levels of the target genes (Fig. 5g), supporting the concept that ZNF354C is a transcriptional repressor of these genes.

### KRAB-A box mutations restore cellular angiogenic sprouting

**Figure 6:**
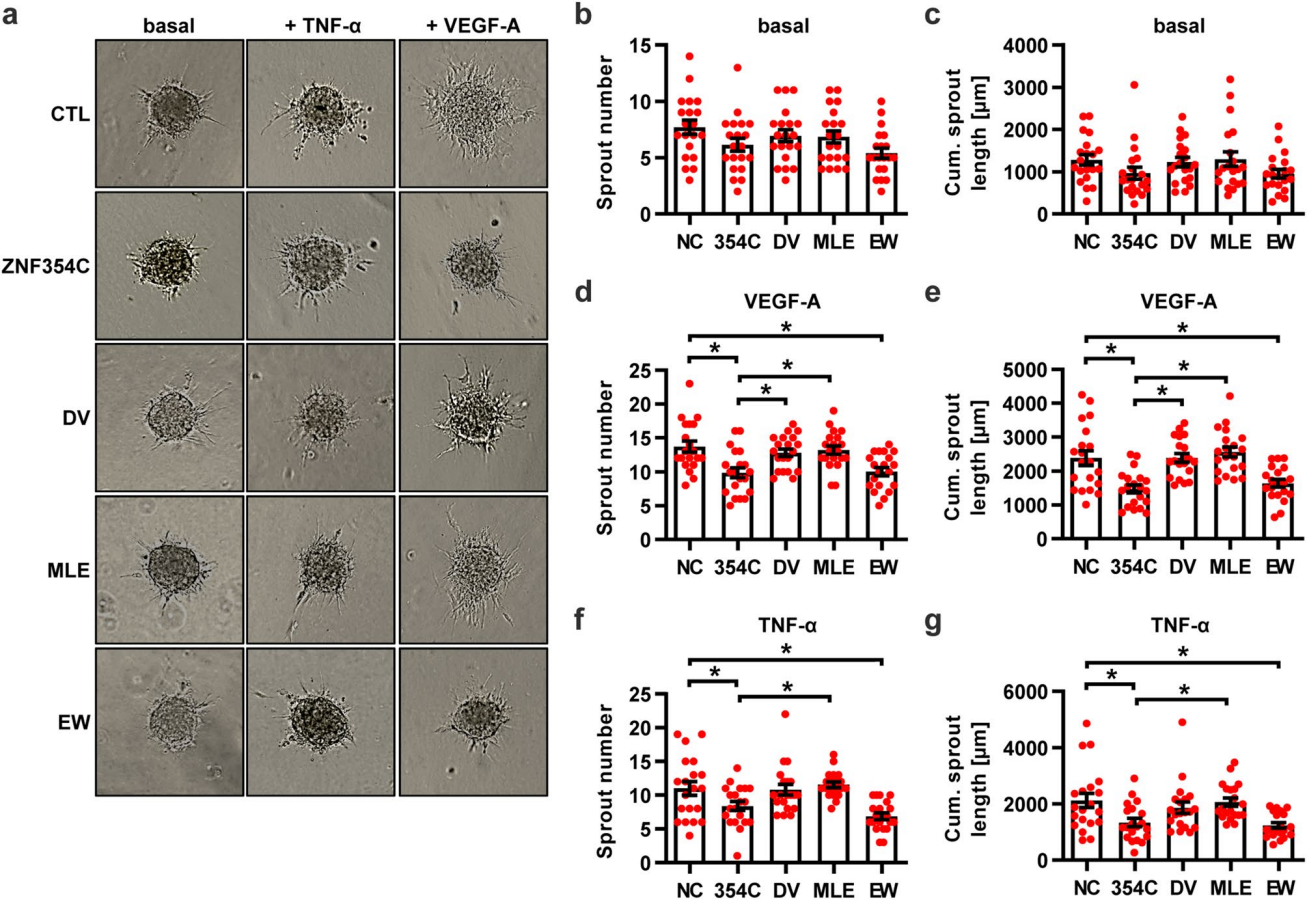
Inhibition of endothelial sprouting by ZNF354C is dependent on the amino acids DV and MLE of the KRAB domain. (**a**–**g**) Spheroid outgrowth assay images (**a**) and quantification of sprout number (**b**, **d**, **f**) and cumulative sprout length (**c**, **e**, **g**) after overexpression of pCMV6-ZNF354C (354C), pCMV6-entry (NC) or the individual mutants DV (DV → AA), MLE (MLE → KKK) or EW (EW → AA). Cells treated under basal conditions (**b**, **c**) or with VEGF-A (**d**, **e**) or TNF-α (**f**, **g**) are shown. Scale bar indicates 50 µm. n = 20. One-Way ANOVA with Bonferroni post-hoc test. *P < 0.05.

Finally, we investigated whether the ZNF354C A-Box KRAB domain mutants have a dominant negative effect on endothelial angiogenic sprouting. Compared to the overexpression of the WT plasmid, the MLE → KKK mutant failed to affect endothelial cell sprouting potential (Fig. 6a–g). A similar effect was observed for the DV → AA mutant, whereas the EW → AA mutant acted as the WT plasmid. None of the constructs, however, induced a higher degree of sprouting than the negative transfection control. This indicates that either the mutants do not exhibit a dominant negative effect or that the endogenous levels of ZNF354C are too low to impact endothelial angiogenic sprouting.

## Discussion

The present study demonstrates that the KRAB-containing C2H2-type zinc finger protein ZNF354C is an important transcription factor that facilitates endothelial cell function. Although ZNF354C is localised to both the nucleus and the cytoplasm, it interacted strongly with dsDNA, TRIM28 and the histone environment. Overexpression of ZNF354C inhibited sprouting angiogenesis in culture. It repressed the transcription of a large number of genes while depletion of ZNF354C with LentiCRISPR or siRNA induced many of these genes. The inhibitory function of ZNF354C was completely dependent on its highly conserved KRAB domain: site-directed mutagenesis of the critical MLE amino acids abolished the repressive effect on gene expression and on endothelial sprouting.

KRAB-domain-containing ZNFs are potent transcriptional repressors^16^. Their inhibitory function is mediated by specific amino acids within the KRAB-A box^16–18^. These amino acids in ZNF354C are highly conserved to other ZNFs and to other mammalian homologues and thus are candidates for the repressive function of ZNF354C. Substitution of the amino acids DV → AA or MLE → KKK not only dramatically reduced the repressive activity of the well-known KOX1 KRAB domain^16^, it also prevented the binding of TRIM28 to the KRAB domain of KOX1^17^. Although the EW sequence has been linked to repression^16^, its role in ZNF354C was minimal. Substitution of MLE → KKK within the KRAB domain of ZNF354C not only abolished the repressive activity but also the inhibition on angiogenic sprouting—substitution of DV → AA was important for angiogenesis but not for transcriptional repression in our settings. This argues that the amino acids MLE within KRAB are probably the most important, potentially mediating the effect through co-repressor binding, as is the case with TRIM28.

In this study, strong differences between the overexpression of ZNF354C in HMEC-1, HUVEC and HAoSMC were observed. Although the overexpression was strongly detectable in HMEC-1, it was very much weaker in HUVECs and not detectable in HAoSMC. Most likely, this is a conseqence of the different transfection efficiencies between the cells. Whereas cell lines as HMEC-1 are usually fairly responsive to transfection, particular HAoSMC are hard to transfect.

The RNA-seq analysis not only confirmed ZNF354C as a transcriptional repressor, it further led to the identification of target genes of ZNF354C. PATL2 is important for mammalian oocyte maturation whose removal causes oocyte meiotic deficiency in humans^21^. HECT and RLD domain containing E3 ubiquitin protein ligase 5 (HERC5) is an E3 ubiquitin ligase which is upregulated by inflammatory cytokines in endothelial cells^22^. HAP1 is involved in intracellular trafficking and linked to Huntington disease^23^. The long non-coding RNA NRAV has been identified to be important for influenza A virus replication through suppression of interferon-stimulated gene transcription^24^. GO analysis revealed that many DEGs are part of cilia/microtubule associated processes, e.g. many Dynein Axonemal Heavy Chain proteins were repressed after ZNF354C overexpression but due to their low expression pattern in the raw RNA-Seq data, these proteins were not further studied.

Also genes relevant for the cardiovascular system were suppressed by ZNF354C: Cardiomyopathy Associated 5 (CMYA5) and Actinin Alpha 2 (ACTN2) are differentially expressed in the myocardium during progression of hypertrophy^25^. Loss of murine RGS5 reduces tumour angiogenesis^20^. This may explain why, after ZNF354C overexpression, sprouting angiogenesis was inhibited.

Taken together, these findings suggest that ZNF354C is important for endothelial cell function by acting as a repressive transcription factor dependent on specific amino acids within its KRAB domain.

## Methods

### Cell culture

All cells were cultured in a humidified atmosphere of 5% CO2 at 37 °C. Cells were cultured similarly as described in^26^. Human microvascular endothelial cells-1 (HMEC-1) (#98247, CDC, Atlanta, GA, USA), pooled human umbilical vein endothelial cells (HUVECs) (#12203, Lot No. 405Z013, 408Z014, 416Z042, PromoCell, Heidelberg, Germany) and human aortic endothelial cells (HAoEC) (#304 K-05a, PeloBiotech, Planegg/Martinsried, Germany) were cultured on fibronectin-coated (#356009, Corning Incorporated, USA) dishes in Endothelial growth medium (endothelial basal medium supplemented with human recombinant epidermal growth factor, EndoCGS-Heparin (PeloBiotech, Planegg/Martinsried, Germany), 8% fetal calf serum (FCS) (#S0113, Biochrom, Berlin, Germany) (for HaoECs 16% FCS), penicillin (50 U/ml) and streptomycin (50 µg/ml) (#15140-122, Gibco/Lifetechnologies, USA)). Human aortic smooth muscle cells (HAoSMC, #354-05a, PeloBiotech, Planegg/Martinsried, Germany), human coronary artery smooth muscle cells (CASMC, #350-05a, PeloBiotech, Planegg/Martinsried, Germany) and human carotid artery smooth muscle cells (CTASMC, #3514-05a, PeloBiotech, Planegg/Martinsried, Germany) were cultured in smooth muscle cell medium classic (#PB-MH-200-2190, PeloBiotech, Planegg/Martinsried, Germany, supplemented with 8% FCS, penicillin (50 U/ml), streptomycin (50 µg/ml), human recombinant epidermal growth factor, fibroblast growth factor, glutamine, and insulin from singlequots (PeloBiotech, Planegg/Martinsried, Germany)). Human embryonic kidney (HEK) 293 cells (ATCC, Manassas, VA, USA) were cultured in Dulbecco's Modified Eagle's Medium (DMEM), high glucose, GlutaMAX (Gibco, Lifetechnologies (Carlsbad, CA, USA)) supplemented with 8% FCS, penicillin (50 U/ml), and streptomycin (50 µg/ml). Human foreskin fibroblasts (Gibco, Lifetechnologies, Carlsbad, CA, USA) were cultured in DMEM/F12 (#11039-021, ThermoFisher, Dreieich, Germany), which was supplemented with 10% FCS, penicillin (50 U/ml) and streptomycin (50 μg/ml). Human monocytic cell line THP-1 and the cancer cell line MCF-7 (both from ATCC, LGC Promochem, Wesel, Germany) were cultured in RPMI medium (with stable glutamine, 8% FCS and 1% penicillin/streptomycin (PAA Laboratories, Cölbe, Germany)).

### Materials and stimulation

The following chemicals and concentrations were used for stimulation in cell culture experiments: Human recombinant VEGF-A 165 (#293-VE, R&D, 50 ng/mL), human recombinant TNF-α (#300-01A, Peprotech, 10 ng/mL) and Cycloheximide (CHX, #C1988, Sigma-Aldrich, 10 µg/mL). For the RNA expression of ZNF354C in 20 different human tissues, the human total RNA master panel II was used (#636643, Clontech/Takara Bio, Saint-Germain-en-Laye, France).

### RNA isolation, reverse transcription and RT-qPCR

Total RNA Isolation was performed with the Quick-RNA MiniPrep kit (Zymo Research, Freiburg, Germany) according to the manufacturer’s protocol. For reverse transcription, SuperScript III Reverse Transcriptase (Thermo Fisher) and oligo(dT)23 together with random hexamer primers (Sigma) were used. CDNA amplification with the oligonucleotides indicated (Table 1) was performed with RT-qPCR using ITaq Universal SYBR Green Supermix and ROX as reference dye (Bio-Rad, #1725125) in an AriaMX cycler (Agilent). Relative expression of human target genes was normalised to b-actin (beta-actin) or 18S ribosomal RNA. Both expressions were analysed with the delta-delta Ct method using the AriaMX qPCR software (Agilent).

**Table 1:** List of primers for qRT-PCR.

Name	Forward primer (5′–3′)	Reverse primer (5′–3′)
b-actin	AAA GAC CTG TAC GCC AAC AC	GTC ATA CTC CTG CTT GCT GAT
ZNF354C	AGG TGA TGC TGG AGA ACT AC	GTC CTG TCT ATG AGG CAA TG
CXCL11	GCC TTG GCT GTG ATA TTG TG	TAT GCA AAG ACA GCG TCC TC
CMYA5	GAG GCA TCC CAA TTC AAA CC	CCT GGC ATT CAG AAG TCA TC
DNAH7	TGT GGC TAA AGC CCT GTA AG	AGG GAA GAG TCA TGG CAA TC
NRAV	CCG CAA CAG ACA CCT GAA AC	TCA GCC TGC CTG TGA GTA AC
DNAH10	CGC CTT GCT TCT TTG TCT AC	GGA CCC ATT GAT TCC GTT TG
CELSR3	AGC CCA CCT CTG GAA TTG TC	CTT TCA CCC GCA CCT CAA AC
RGS5	GGA TTG CCT GTG AGG ATT AC	TCT TTA GGA GCC TCC GTT TG
CFAP57	GTT CAC CTG GAA GGT CTT TG	TCC TCC ACA CGA GTC TTT AG
WDR78	TAT GGA ACG GGT TCT GAT GG	CAA GTT GGC GGG TAT TGT TG
HERC5	GCT GCG TCA TAT TCA GTC AC	TGC TGC CGA CCT AAG ATA AG
BSN	CAG CAC GGG AAG TTA TGG TC	TGG AGG CAG GCA ATG TAG AG
DNAH12	AGA ATG CCC GAG TTC GTA TC	AGG CGT AAT GAC AAG TCG AG
HAP1	GGA GAC TCT TCC TGG TTT CC	GCT CTC GAT CCT CAC TGT AG
CFAP43	GCC TGG AAT GGA TGT GAA CC	CAC CGA CCT TGC TCT GAA AC
PATL2	CAA CTG GTA TCG CTG CAT TC	TAG GCA TTT GGG CTA TCT CC
HSPA6	CCA AGC AGA CCC AGA CTT TC	CAC GCT CAG GAT GCC ATT AG

### Knockdown and overexpression procedures

For small interfering RNA (siRNA) treatments, HMEC-1 were transfected with Lipofectamine RNAiMAX and HUVECs were transfected with GeneTrans II according to the instructions provided (Lipofectamine RNAiMAX: Thermo Fisher Scientific, Waltham (USA); GenetransII: MoBiTec (Göttingen, Germany). HAoSMC were transfected with Lipofectamine 3000 according to the manufacturer’s protocol (Thermo Fisher Scientific, Waltham, USA). ZNF354C stealth siRNAs (siZNF354C_1 as HSS121243, siZNF354C_2 as HSS179215, #1299001) were from Invitrogen (Carlsbad, USA). As a negative control, scrambled Stealth RNAi Med GC (Invitrogen, #12935300) was used. If not otherwise indicated, siRNA experiments were performed for 48–72 h.

Overexpression of C-terminally Myc-DDK-tagged pCMV6-ZNF354C (NM_014594, #RC209312, Origene), pCMV6-ZNF354C_DV, pCMV6-ZNF354C_MLE or pCMV6-ZNF354C_EW in HUVECs, HMEC-1 or HAoSMCs was performed by electroporation with the Neon Transfection System (ThermoFisher), according to the instructions provided. The pCMV6-entry vector (#PS100001, Origene) served as negative control. HAoSMC were seeded on collagen-coated dishes. 24 h after electroporation the cells were used for further experiments.

### Protein isolation, western analysis and nuclear-cytoplasmic extraction

HUVECs were washed in Hanks solution (Applichem). After cell lysis with Triton X-100 buffer (20 mM Tris/Cl pH 7.5, 150 mM NaCl, 10 mM NaPPi, 20 mM NaF, 1% Triton, 2 mM Orthovanadat, 10 nM Okadaic Acid, protein-inhibitor mix, 40 µg/ml Phenylmethylsulfonylfluorid), the extract was centrifuged (10 min, 16,000×*g*, 4 °C). The protein concentration of the supernatant was determined with the Bradford assay. The cell extract was boiled in Laemmli buffer and equal amounts of protein were separated with SDS-PAGE. The gels were blotted onto a nitrocellulose membrane and blocked in Rotiblock (Carl Roth, Germany). After incubation with the first antibody, infrared-fluorescent-dye-conjugated secondary antibodies (Licor, Bad Homburg, Germany) were used and signals detected with an infrared-based laser scanning detection system (Odyssey Classic, Licor, Bad Homburg, Germany). Antibodies against beta-actin (b-actin, #A1978, Sigma-Aldrich, Germany), ZNF354C (#NBP1-81352, Novus Biologicals, Cenntenial (USA)), Flag (#F3165-0.2MG, Sigma-Aldrich, Germany), Topoisomerase I (#sc-32736, Santa Cruz Biotechnology, Dallas (USA)) and HSC70/HSP70 (#ADI-SPA-820, Enzo Life Sciences, Lörrach, Germany) were used.

For fractionation experiments, cells were treated with buffer A (10 mM HEPES pH 7.9, 10 mM KCl, 0.1 mM EDTA, 0.1 mM EGTA, 0.75% Nonidet, 2 mM Orthovanadat, 10 nM Okadaic Acid, protein-inhibitor mix, 40 µg/ml Phenylmethylsulfonylfluorid) for 15 min on ice. The lysate was vortexed and centrifuged (1 min, 16,000×*g*, 4 °C). The supernatant was used as cytoplasmic extract, whereas the pellet was washed in buffer A and centrifuged (1 min, 16,000×*g*, 4 °C). The pellet was further treated with buffer C (20 mM HEPES pH 7.9, 0.4 M NaCl, 1 mM EDTA, 1 mM EGTA, 2 mM Orthovanadat, 10 nM Okadaic Acid, protein-inhibitor mix, 40 µg/ml Phenylmethylsulfonylfluorid) for 15 min on ice, centrifuged (5 min, 16,000×*g*, 4 °C) and the supernatant was used as nuclear extract.

### Immunoprecipitation

Nuclei isolation of 10^7^ HMEC-1 per sample was performed as described above with buffer A (10 mM HEPES pH 7.9, 10 mM KCl, 0.1 mM EDTA, 0.1 mM EGTA, 0.75% Nonidet, 2 mM Orthovanadat, 10 nM Okadaic Acid, protein-inhibitor mix, 40 µg/ml Phenylmethylsulfonylfluorid) followed by buffer C (20 mM HEPES pH 7.9, 0.4 M NaCl, 1 mM EDTA, 1 mM EGTA, 2 mM Orthovanadat, 10 nM Okadaic Acid, protein-inhibitor mix, 40 µg/ml Phenylmethylsulfonylfluorid), each step with 15 min incubation at 4 °C. After pre-clearing of the supernatant with 20 µL DiaMag Protein A and Protein G beads (Diagenode), the lysed nuclei were incubated with H3-pan (#C15200011, Diagenode), H3K4me3 (#C15410003, Diagenode) or dsDNA [35I9 DNA] (#ab27156, Abcam) antibodies overnight at 4 °C. The samples were then incubated with 50 µl of total DiaMag Protein A and Protein G (Diagenode) beads for 2 h at 4 °C, followed by 3 washing steps in buffer C. Prior to elution, beads were put into a new Eppendorf tube, boiled in Laemmli buffer and western analyses was performed.

### mRNA-sequencing, bioinformatics and data deposition

For RNA-seq analysis, total RNA was isolated from HMEC-1 with Quick-RNA MiniPrep kit (Zymo Research, Freiburg, Germany) according to the manufacturer’s protocol. RNA and library preparation integrity were verified with LabChip Gx Touch 24 (Perkin Elmer). 2 µg of total RNA was used as input for VAHTS Stranded mRNA-seq Library preparation following manufacturer’s protocol (Vazyme). Sequencing was performed on NextSeq500 instrument (Illumina) using v2 chemistry, resulting in an average of 21Mio reads per library with 1 × 75 bp single end setup. The resulting raw reads were assessed for quality, adapter content and duplication rates with FastQC^27^. Trimmomatic version 0.39 was employed to trim reads after a quality drop below a mean of Q20 in a window of 5 nucleotides^28^. Only reads between 30 and 150 nucleotides were cleared for further analyses. Trimmed and filtered reads were aligned against the Ensembl human genome version hg38 (GRCh38) using STAR 2.6.1d with the parameter “–outFilterMismatchNoverLmax 0.1” to increase the maximum ratio of mismatches to mapped length to 10%^29^. The number of reads aligning to genes was counted with featureCounts 1.6.5 tool from the Subread package^30^. Only reads mapping at least partially inside exons were admitted and aggregated per gene. Reads overlapping multiple genes, or aligning to multiple regions, were excluded. Differentially expressed genes were identified using DESeq2 version 1.18.1^31^. Only genes with a minimum fold change of log2 =  ± 0.05, a maximum Benjamini–Hochberg corrected p-value of 0.05, and a minimum combined mean of 5 reads were deemed to be significantly differentially expressed. The Ensemble annotation was enriched with UniProt data (release 06.06.2014) based on Ensembl gene identifiers^32^.

Gene ontology terms were calculated with the PANTHER (Protein ANalysis THrough Evolutionary Relationships, https://geneontology.org/) online tool^33^ under default conditions. The analysis type was PANTHER overrepresentation test (release date 20200728). For PANTHER protein class determination, the annotation version and release date was PANTHER version 15.0, released 2020-02-14. For PANTHER GO determination, the annotation version and release date was GO Ontology database (GO data archive 10.5281/zenodo.3954044, Released 2020-07-16). For both, *Homo sapiens* was chosen as reference list with the test type Fisher’s exact and the false discovery rate was selected as correction.

The dataset has been deposited and is available at GEO datasets with the GEO accession number GSE155270 (https://www.ncbi.nlm.nih.gov/geo/query/acc.cgi?acc=GSE155270).

### Immunofluorescence

Cells were seeded on 8-well immunofluorescence plates (Ibidi). After incubation for a further 24 h to reach 80% confluency, cells were washed with PBS, fixed with 4% paraformaldehyde, quenched with glycine (2%), washed again with PBS and permeabilised with 0.05% Triton X-100. After blocking with 3% BSA for 30 min, the cells were incubated at 4 °C overnight with a 1:200 dilution of the primary antibody against anti-Flag M2-Cy3 (#A9594-0.2MG; Sigma-Aldrich, Germany) or ZNF354C (#NBP1-81352, Novus Biologicals, Cenntenial (USA)). Cells were washed with 0.3% Tween20 in PBS and incubated with a 1:500 dilution of secondary antibody (Rabbit IgG (Alexa Fluor 647, #A31573, Invitrogen, Carlsbad, USA) for 30 min. For staining with the fluorescent primary antibody anti-Flag M2-Cy3 (#A9594-0.2MG; Sigma-Aldrich, Germany), treatment was performed for 1 h at RT without applying a secondary antibody. Finally, four washing steps were performed: 1 × with PBS (0.3% Tween, 0.1 mg/ml DAPI, 5 min), 2 × with PBS (0.3% Tween, 3 min) and 1 × with pure PBS for 3 min. For the detection of two proteins within one chamber, antibody staining was performed with two primary antibodies originating from different species. Cells were then washed again with 0.3% Tween and counterstained with DAPI. Images were captured with a laser confocal microscope LSM800 (Zeiss, Germany) and analysed with ZEN lite software (Zeiss, Germany).

### Proximity ligation assay (PLA)

The PLA was performed as described in the manufacturer’s protocol (Duolink II Fluorescence, OLink, Upsalla, Sweden). HMEC-1 were fixed in phosphate buffered formaldehyde solution (4%), permeabilised with Triton X-100 (0.2%), blocked with serum albumin solution (3%) in phosphate-buffered saline, and incubated overnight with the following antibodies: ZNF354C (#NBP1-81352, Novus Biologicals, Cenntenial (USA)), dsDNA antibody [35I9 DNA] (#ab27156, Abcam) or TIF1β (D-7, TRIM28) (#sc-515790, Santa Cruz Biotechnology). Samples were washed and incubated with the respective PLA-probes for 1 h at 37 °C. After washing, samples were ligated for 30 min (37 °C). After an additional washing step, the amplification with polymerase was performed for 100 min (37 °C). The nuclei were stained using DAPI. Images (with Alexa Fluor, 546 nm) were acquired by confocal microscope (LSM 510, Zeiss).

### Spheroid outgrowth assay

Spheroid outgrowth assays were performed as described in^34^. HMEC-1 spheroids were stimulated for 16 h with human recombinant VEGF-A 165 (293-VE, R&D, 50 ng/ml) or human recombinant TNF-α (300-01A, Peprotech, 10 ng/ml). Images were generated with the Evos XL Core (ThermoFisher). The quantitative analysis of sprout number and cumulative length was calculated with the AxioVision software (Zeiss).

### Chromatin immunoprecipitation (ChIP)

HMEC-1 were electroporated with pCMV6-entry (#PS100001, Origene) or pCMV6-ZNF354C (NM_014594, #RC209312, Origene) and incubated for 24 h. Crosslinking and isolation of nuclei was performed with the truCHIP Chromatin Shearing Kit (Covaris, USA) according to the manufacturer’s protocol. ChIP was performed as described in^35^. Samples were incubated overnight at 4 °C with a monoclonal anti-Flag M2 antibody (F3165-0.2MG, Sigma-Aldrich). 5% of the samples served as input. After elution, the DNA was purified with the QiaQuick PCR purification kit (Qiagen, Hilden, Germany) and subjected to qPCR analysis. As a negative control during qPCR, oligonucleotides targeting the GAPDH promoter were used. The primers are listed in Table 2.

**Table 2:** List of promoter primers for ChIP.

Name	Forward primer (5′–3′)	Reverse primer (5′–3′)
RGS5	CAG TAG TGC CTG TAG CAG AG	GCC AAT CCA GAG CCT TAG AG
PATL2	GCT CAC ATG ACC CGG TGA AG	GGA AAG TCC TGG GTA GAT CC
HAP1	ATG ACC CCA GCT CAC CAA GG	CCA GGC AAA GAC TGA GGA CA
NRAV	AAA TAA GGC AGC GAG GAC AC	GTA GCG ACG GTA TCT CTA GC
ADAMTS1	TTC GGT TGG AGA ACG CAG TCC	GAA GGT GGA GAA GTG GGG TGA G

### Site-directed mutagenesis

Three individual KRAB domain mutations (a. D17A/V18A (DV); b. M41K/L42K/E43K (MLE), c. E25A/W26A (EW)) of pCMV6-ZNF354C were generated with the Q5 Site-Directed Mutagenesis Kit (#E0554S, NEB) according to the instructions of the manufacturer. To generate primer sequences and an annealing temperature, the NEBaseChanger (NEB) was used. The pCMV6-ZNF354C plasmid served as template and was amplified with PCR (Anneling temperature 66 °C) with the following oligonucleotides to obtain the individual mutants: DV, 5′-GAC ATT CAG Ggc tgc gGC CGT GTT CTT-3′ and 5′-ACA GGC TCC TGA GCA GAC-3′, MLE 5′-gaa gAA CTA CAG CAG CCT GGT C-3′ and 5′-ttc ttC ACC TCC CGG TAC AAG GC-3′, EW 5′-CAG CCA GGA Cgc ggc gTT GCA CCT GG-3′ and 5′-AAG AAC ACG GCC ACA TCC-3′. The final plasmids were verified by sequencing.

### LentiCRISPRv2

Guide RNAs (gRNA) targeting ZNF354C were selected using the publicly available CRISPOR algorithm (https://crispor.tefor.net/)^36^. The gRNAs 1 and 3 targeted the TSS, gRNA2 targeted a region upstream of the TSS and gRNAs 4–6 targeted a region dowstream of the TSS. The gRNAs were cloned into lentiCRISPRv2 vector backbone with BsmBI according to the standard protocol^37^. LentiCRISPR v2 was a gift from Feng Zhang (Addgene plasmid #52961). The following oligonucleotides were used for annealing: (1) 5′-CAC CGA GTG GTA GTT CTC CCG CGG T-3′ and 5′-AAA CAC CGC GGG AGA ACT ACC ACT C-3′, (2) 5′-CAC CGA GTA CGC ACG CCG CAC ACG-3′ and 5′-AAA CCG TGT GCG GCG TGC GTA CTC-3′, (3) 5′-CAC CGG TAG TTC TCC CGC GGT TGG-3′ and 5′-AAA CCC AAC CGC GGG AGA ACT ACC-3′, (4) 5′-CAC CGC AAG GGG CCC GAG CCG CGT A-3′ and 5′-AAA CTA CGC GGC TCG GGC CCC TTG C-3′, (5) 5′-CAC CGC CAA GGG GCC CGA GCC GCG T-3′ and 5′-AAA CAC GCG GCT CGG GCC CCT TGG C-3′, (6) 5′-CAC CGA CGC CGC CCA CTC GCT GGA G-3′ and 5′-AAA CCT CCA GCG AGT GGG CGG CGT C-3′. After cloning, the gRNA-containing LentiCRISPRv2 vectors were purified and sequenced. Lentivirus was produced in Lenti-X 293 T cells (#632180, Takara) using Polyethylenamine (#408727, Sigma-Aldrich), psPAX2 and pVSVG (pMD2.G). psPAX2 and pMD2.G were a gift from Didier Trono (Addgene plasmid #12260; Addgene plasmid #12259). LentiCRISPRv2-produced virus was transduced in HMEC-1 with polybrene transfection reagent (MerckMillipore, # TR-1003-G) and selection was performed with puromycin (1 μg/ml) for 8 days prior to RNA and genomic DNA isolation.

### Isolation of genomic DNA

For gDNA isolation, cell pellets were washed with Hank’s buffer and lysed in proteinase K-containing lysis buffer. After 2 h of incubation (800 rpm, 56 °C), cells were centrifuged (13,200 rpm, 1 min, RT) and the supernatant was mixed 1:1 with isopropanol. Precipitated DNA was centrifuged down (13,200 rpm, 20 min, 4 °C), resuspended in ethanol (70%), centrifuged (13,200 rpm, 10 min, 4 °C) and the supernatant was aspirated and DNA isolated and dissolved in TE buffer. The samples were further subjected to PCR with PCR master mix solution (#K0171, ThermoFisher) containing Taq DNA Polymerase and primers targeting the ZNF354C TSS (FP: 5′-CAC ACT GAT CCT CCT AAA TG-3′, RP: 5′-CAA CGA CAA AAG GCT GGC ATC-3′). The expected length of the PCR product in NTC-treated samples was 628 nt. GAPDH primer (FP: 5′-TGG TGT CAG GTT ATG CTG GGC CAG-3′, RP: 5′-GTG GGA TGG GAG GGT GCT GAA CAC-3′; expected size: 128 nt) served as loading control.

### ZNF354C domain annotation and publicly available datasets used

Domain annotations were from UniProt^38^. Diagrams were constructed using Domain Graph (DOG), version 2.0^39^. Fantom5 Encode CAGE expression data was obtained from FANTOM5 website (gencode v19) and only q20_tpm values of CellPapAIn (Whole cell, Poly(A) + RNA longer than 200 nt ) replicates 1 and 2 were used^40–42^.

### Statistics

Unless otherwise indicated, data are provided as means ± standard error of mean (SEM). Calculations were performed with GraphPad Prism (Version 8.4.3 (646)) or BiAS.10.12. The latter was also used to test for normal distribution and similarity of variance. In the case of multiple testing, Bonferroni correction was applied, with the exception of RNA-seq, for which Benjamini Hochberg correction was applied. For multiple group comparisons ANOVA followed by post hoc testing was performed. Individual statistics of dependent samples were performed with paired t-test, of unpaired samples by unpaired t-test and if not normally distributed by Mann–Whitney test. P values of < 0.05 were considered significant. Unless otherwise indicated, n indicates the number of individual experiments.

## Supplementary information

Supplementary Information.

Supplementary Table S1.
