# Supplementary material for: ZNF354C is a transcriptional repressor that inhibits endothelial angiogenic sprouting
Source: Sci Rep. 2020 Nov 5;10:19079. doi: 10.1038/s41598-020-76193-0 (PMC7645770; doi:10.1038/s41598-020-76193-0)
Supplement: Supplementary file 1 — Supplementary Information. [file 41598_2020_76193_MOESM1_ESM.pdf]

Supplementary Information

ZNF354C is a transcriptional repressor that inhibits endothelial angiogenic sprouting

James A. Oo[1,3], Barnabas Irmer[1], Stefan Günther[2], Timothy Warwick[1], Katalin Pálfi[1], Judit Izquierdo Ponce[1], Tom Teichmann[1], Beatrice Pflüger-Müller[1,3], Ralf Gilsbach[1,3], Ralf P. Brandes[1,3], Matthias S. Leisegang[1,3]*

[1] Institute for Cardiovascular Physiology, Goethe University, Frankfurt, Germany

[2] Max Planck Institute for Heart and Lung Research, Bad Nauheim, Germany

[3] German Center of Cardiovascular Research (DZHK), Partner site RheinMain, Frankfurt, Germany

* Corresponding author

Matthias S. Leisegang, PhD

Institut für Kardiovaskuläre Physiologie

Fachbereich Medizin der Goethe-Universität

Theodor-Stern-Kai 7

60590 Frankfurt am Main, Germany

Tel.: +49-69-6301-6996

Fax.: +49-69-6301-7668

Email: Leisegang@vrc.uni-frankfurt.de

Supplementary information file contains

- Supplementary figure 1
- Supplementary figure 2
- Supplementary figure 3
- Uncropped western blots

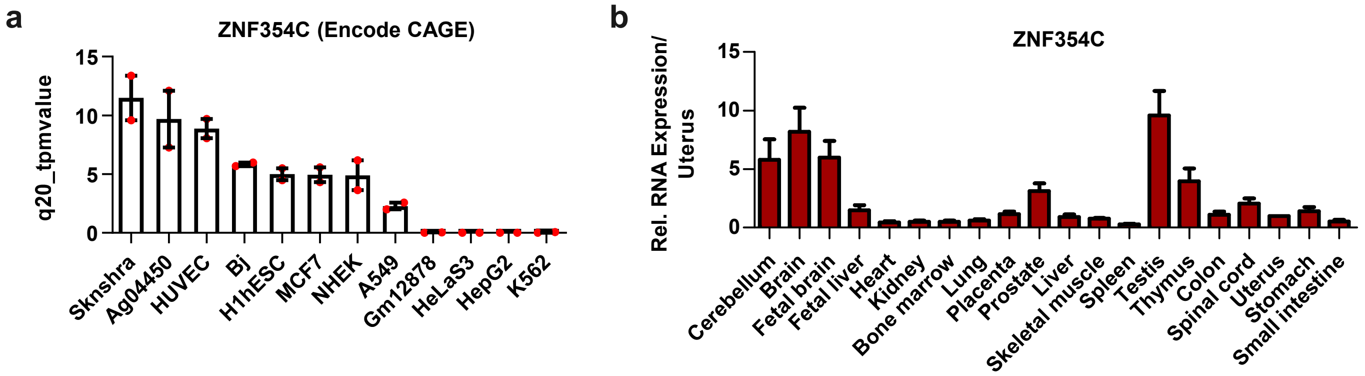

Supplementary Figure 1: (**a**) Encode FANTOM5 Cap Analysis of Gene Expression data of ZNF354C in representative cell types (whole cell). Q20 transcripts per million is shown on the Y-axis. n=2. Data are mean ± SEM. (**b**) Expression of ZNF354C in 20 different human tissues measured by RT-qPCR relative to Uterus expression, which was set to 1. n=3.

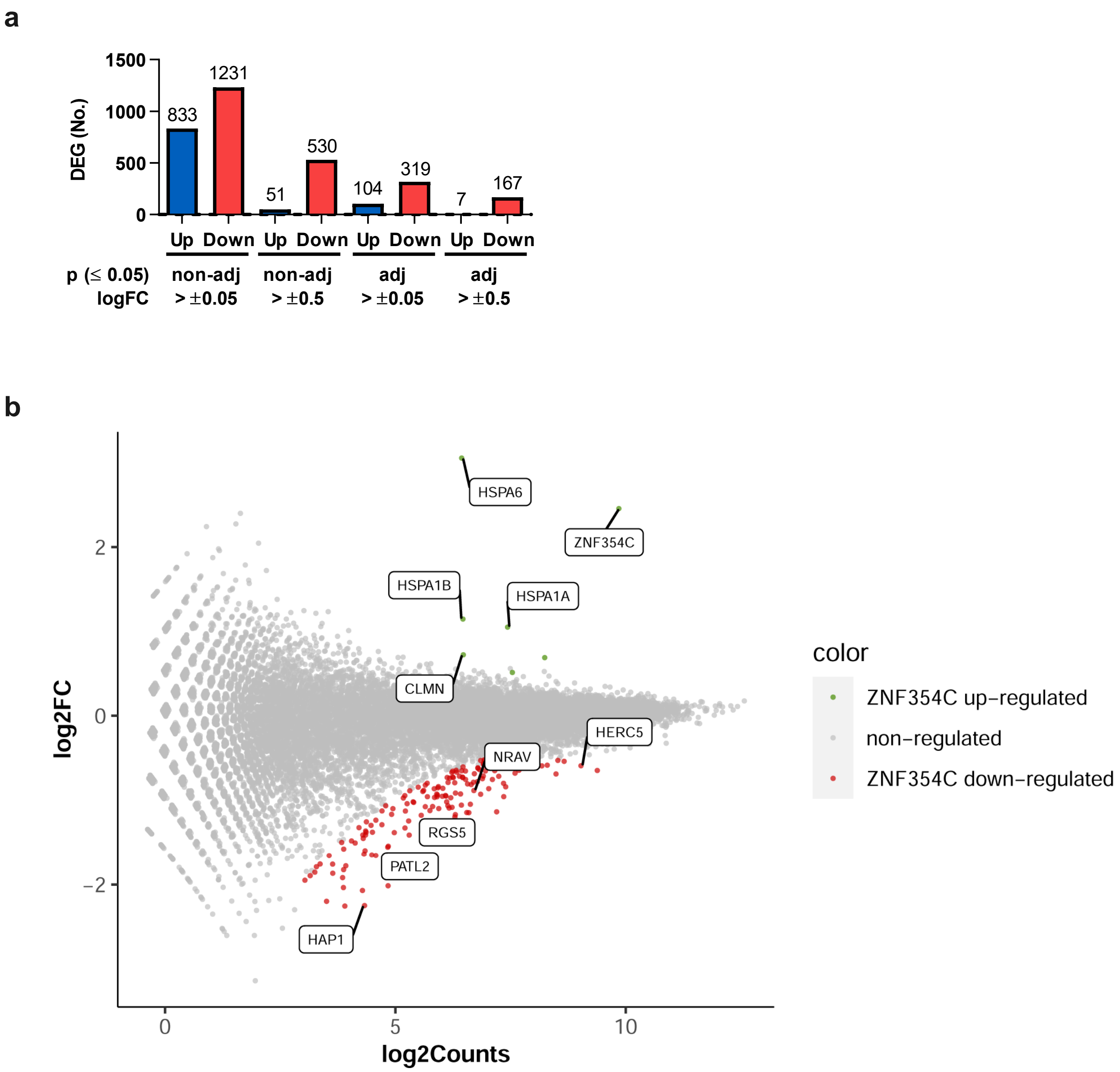

Supplementary Figure 2: (**a**) Number of differentially expressed genes (DEG) of the RNA-Seq of up- or downregulated genes after ZNF354C overexpression. Conditions are indicated (adjusted or non-adjusted p-value<0.05; logFC>±0.05 or >±0.5). (**b**) MA plot of RNA-Seq after overexpression of pCMV6-ZNF354C (ZNF354C) or pCMV6-Entry (CTRL). Conditions are adjusted p-value<0.05 (FDR); Colored points indicate genes significantly differentially expressed (log2FC>±0.5). Red, ZNF354C downregulated genes; green, ZNF354C upregulated genes; grey, non-regulated genes (log2FC<±0.5).

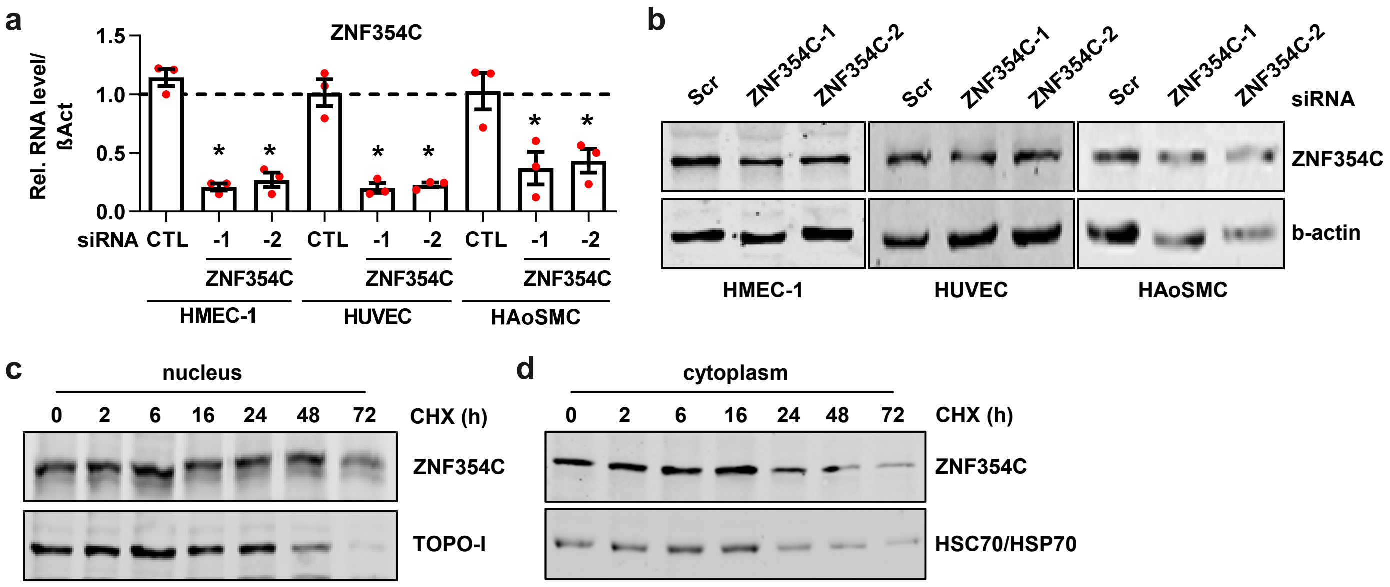

Supplementary Figure 3: (**a**) RT-qPCR of ZNF354C after transfection of HMEC-1, HUVEC or HAoSMC with siRNAs targeting ZNF354C (-1, -2). Scrambled siRNA (CTL) served as negative control. n=3. Data are mean ± SEM. Mann-Whitney t-test; *P<0.05. (**b**) Western blot after transfection of HMEC-1, HUVEC or HAoSMC with siRNAs targeting ZNF354C (-1, -2). Scrambled siRNA (CTL) served as negative control. ZNF354C and b-actin antibodies were used. (**c&d**) Western blot after fractionation of HUVEC into nucleus (c) and cytoplasm (d) and treatment with Cycloheximide (CHX) for the indicate time (h). ZNF354C, Topoisomerase I (Topo-I) and HSC70/HSP70 antibodies were used.

Fig. 1B

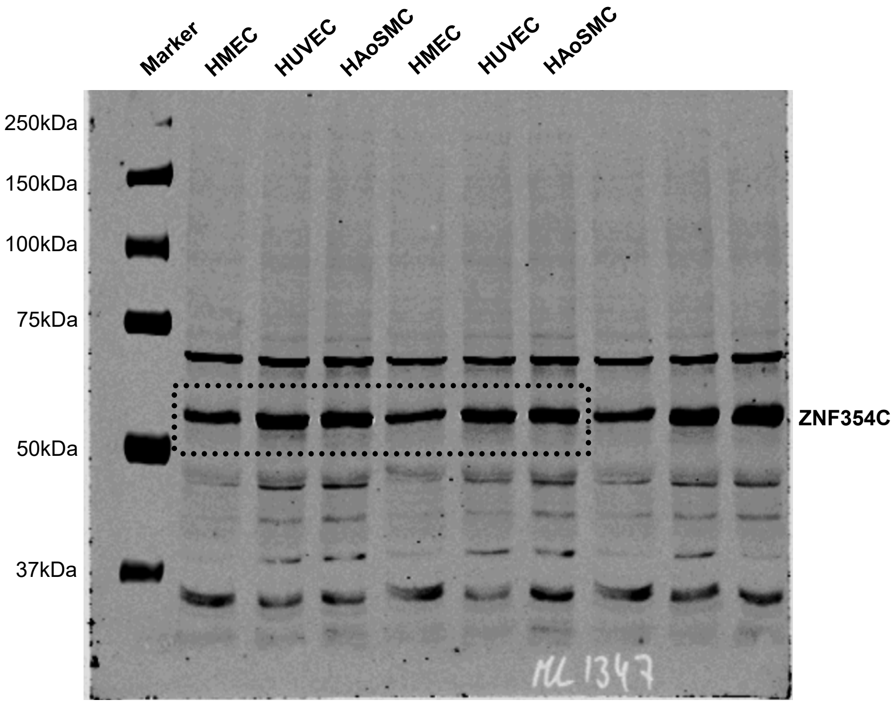
Marker
HMEC
HUVEC
HAoSMC
HMEC
HUVEC
HAoSMC
250kDa
150kDa
100kDa
75kDa
50kDa
37kDa
ZNF354C

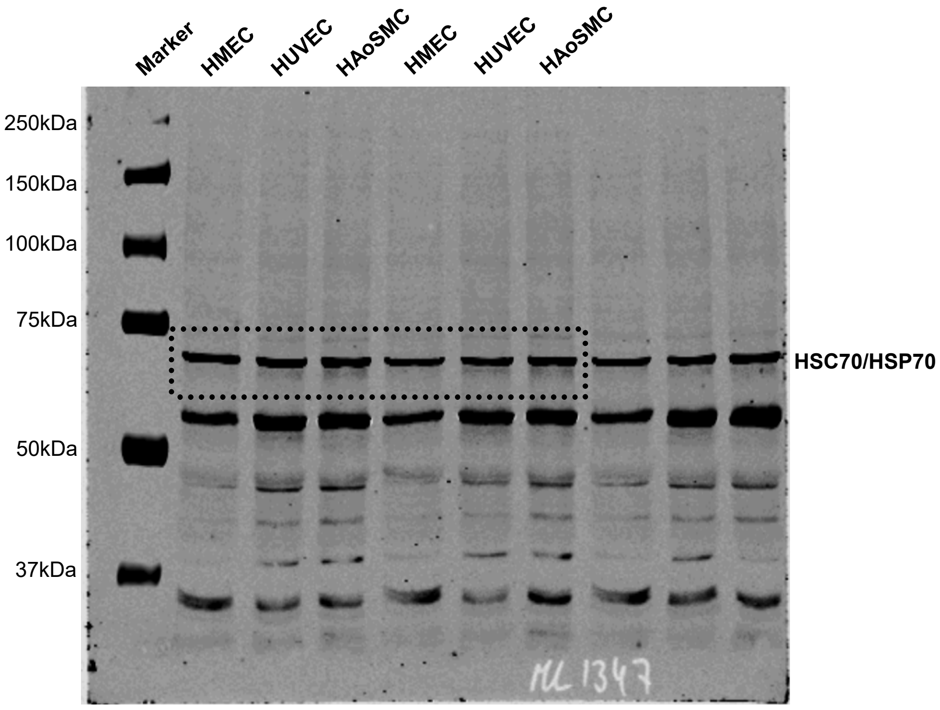
Marker
HMEC
HUVEC
HAoSMC
HMEC
HUVEC
HAoSMC
250kDa
150kDa
100kDa
75kDa
50kDa
37kDa
HSC70/HSP70

Fig. 1E (Left: HMECs)

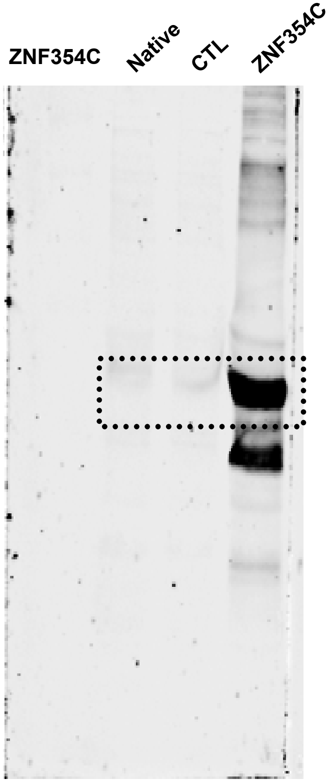

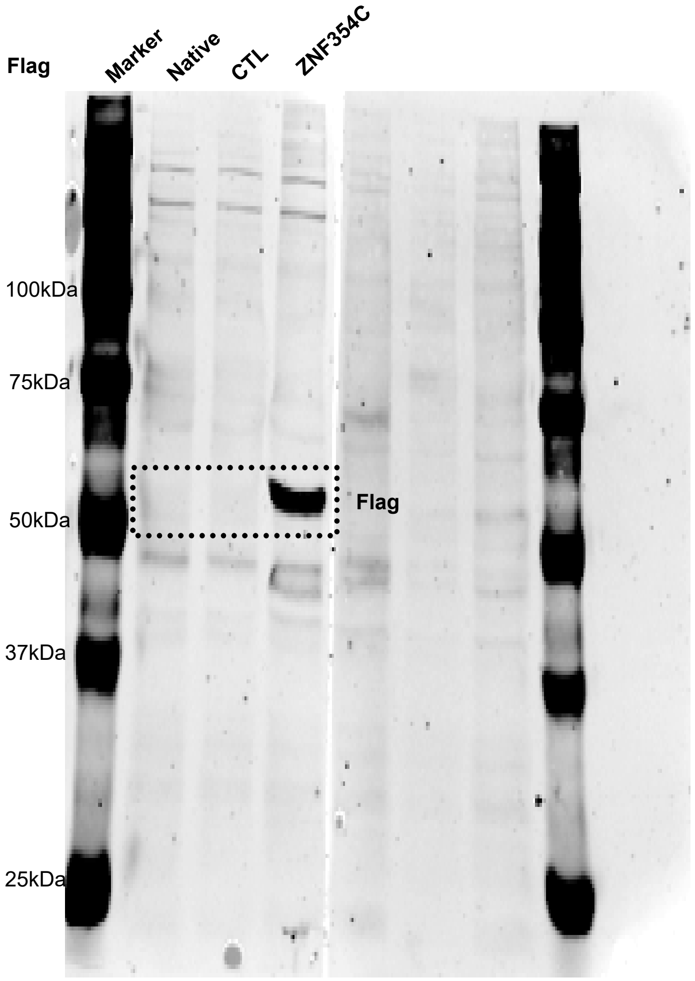

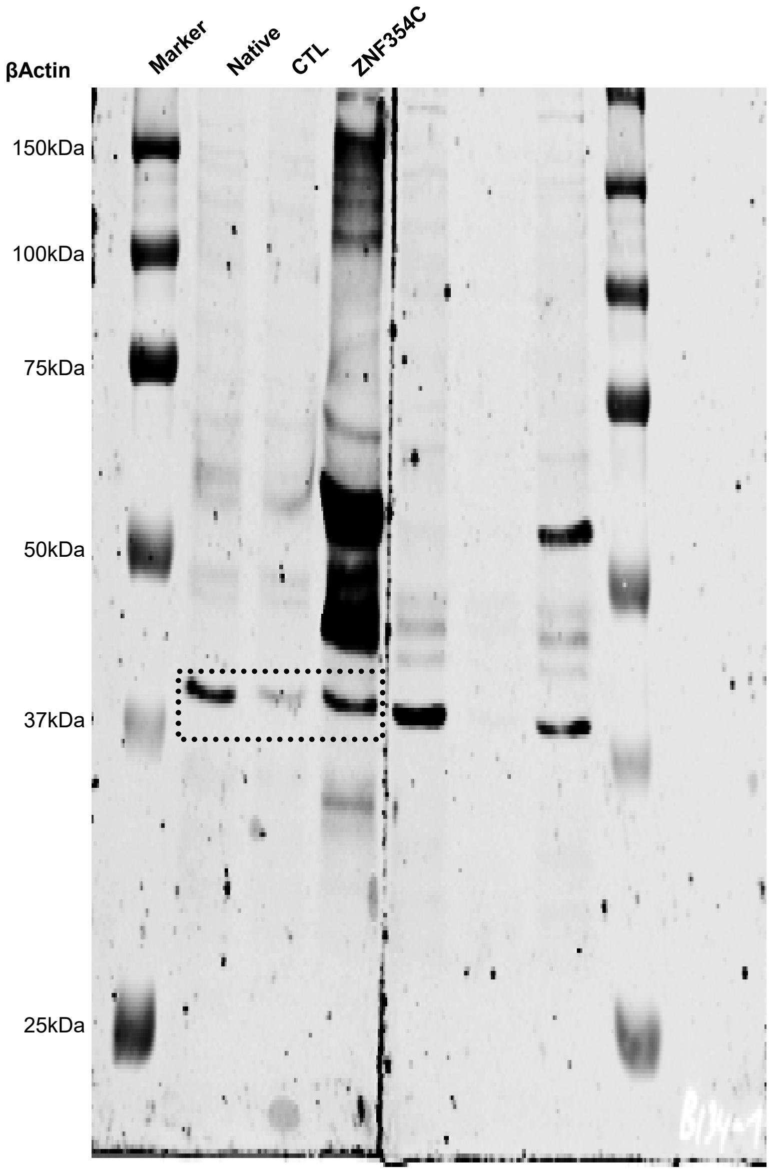

Fig. 1E (Middle: HUVECs)

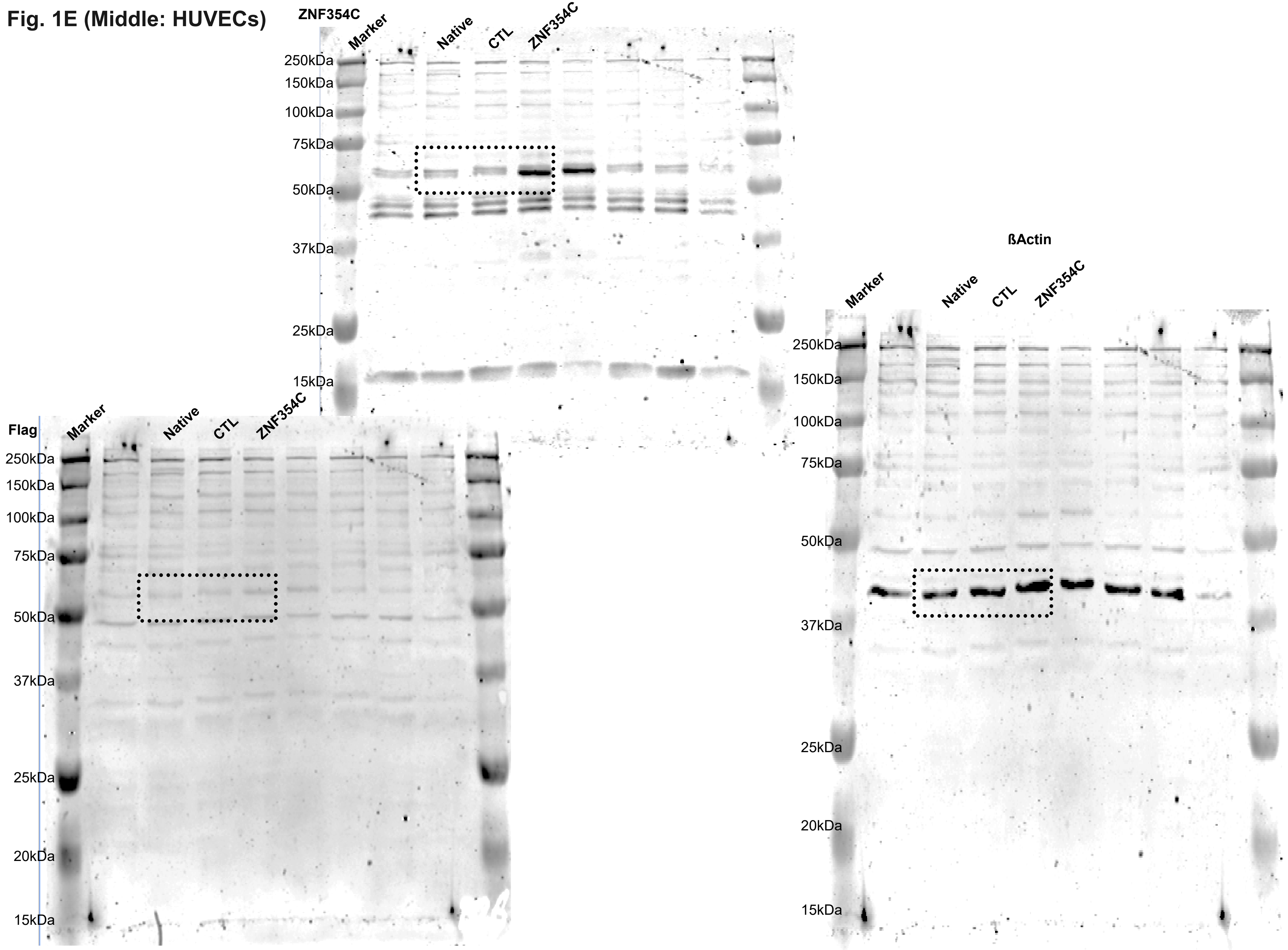

Fig. 1E (Right: HAoSMCs)

ZNF354C

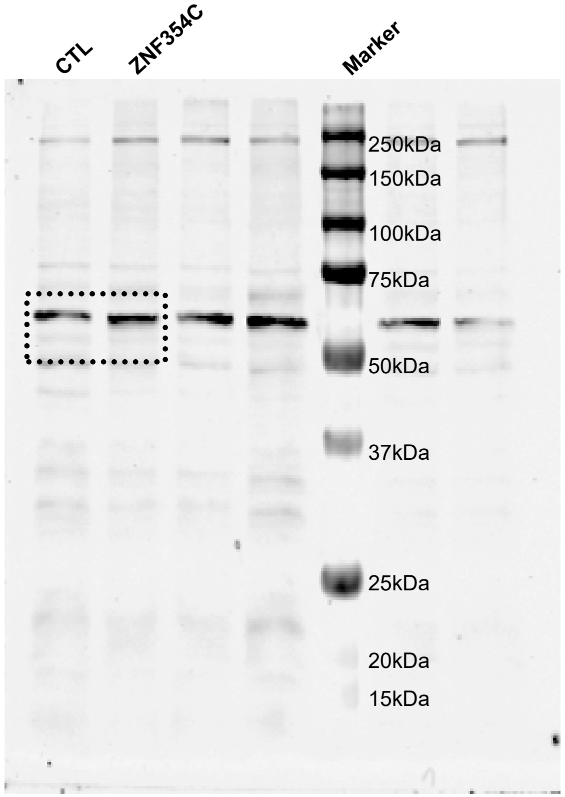

Flag

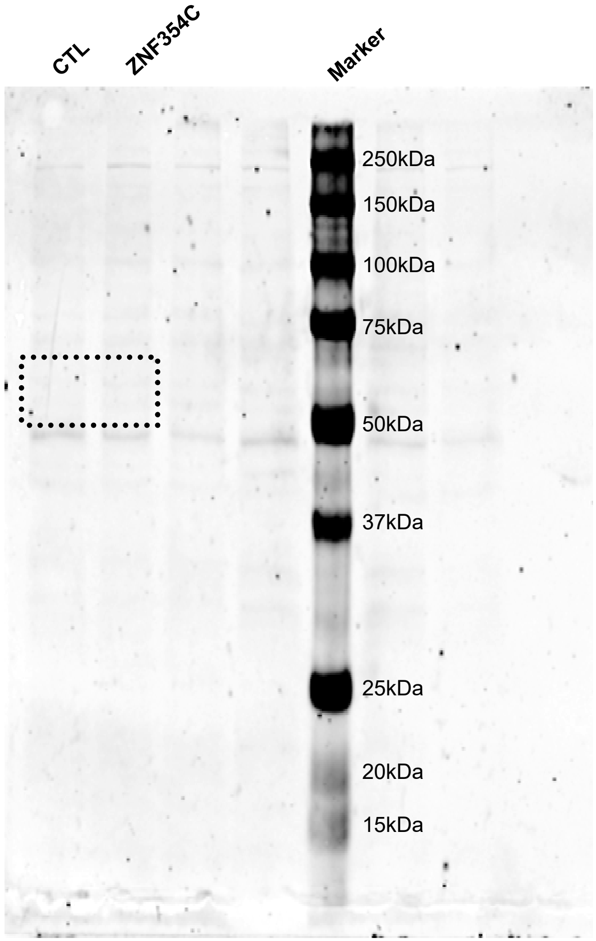

βActin

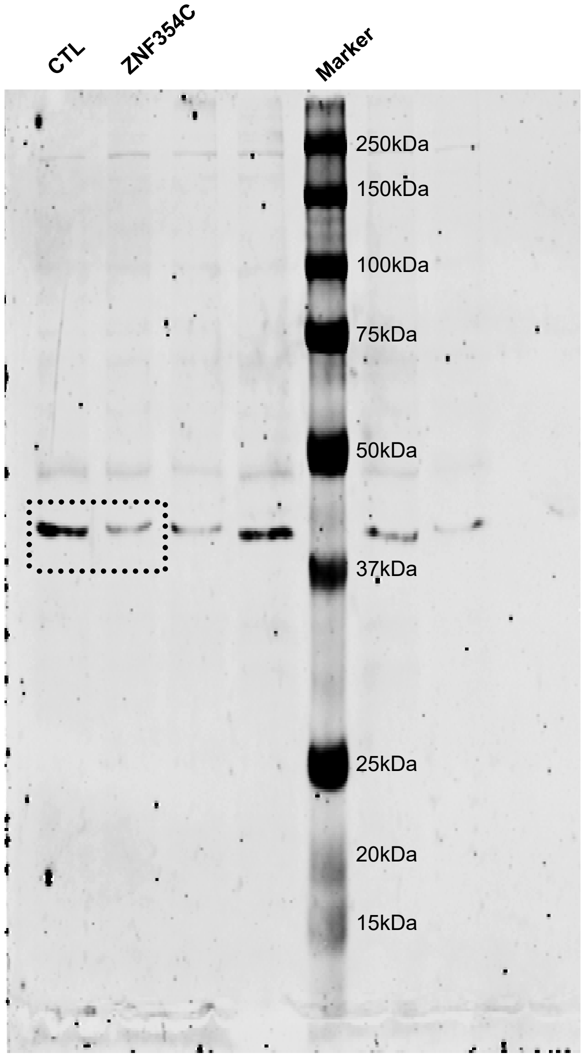

Fig. 3E

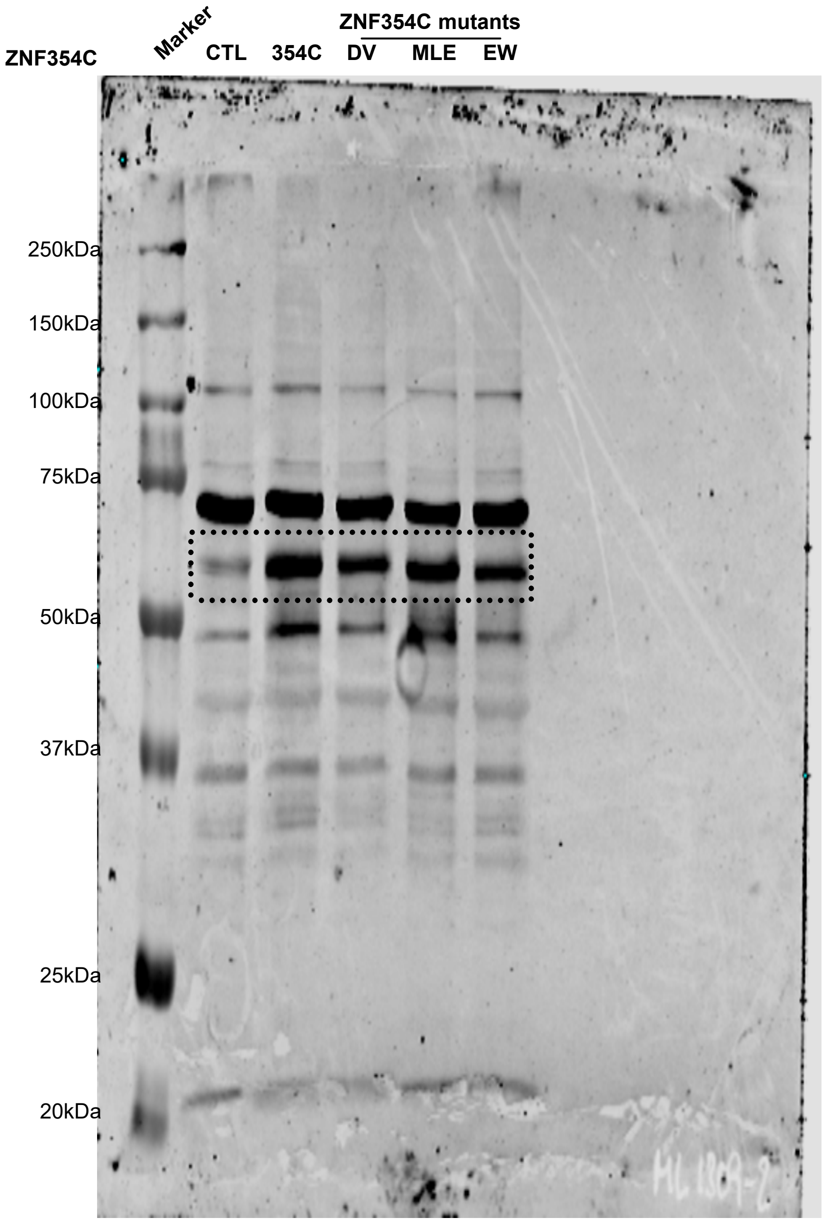
ZNF354C
Marker
CTL
354C
ZNF354C mutants
DV
MLE
EW
250kDa
150kDa
100kDa
75kDa
50kDa
37kDa
25kDa
20kDa

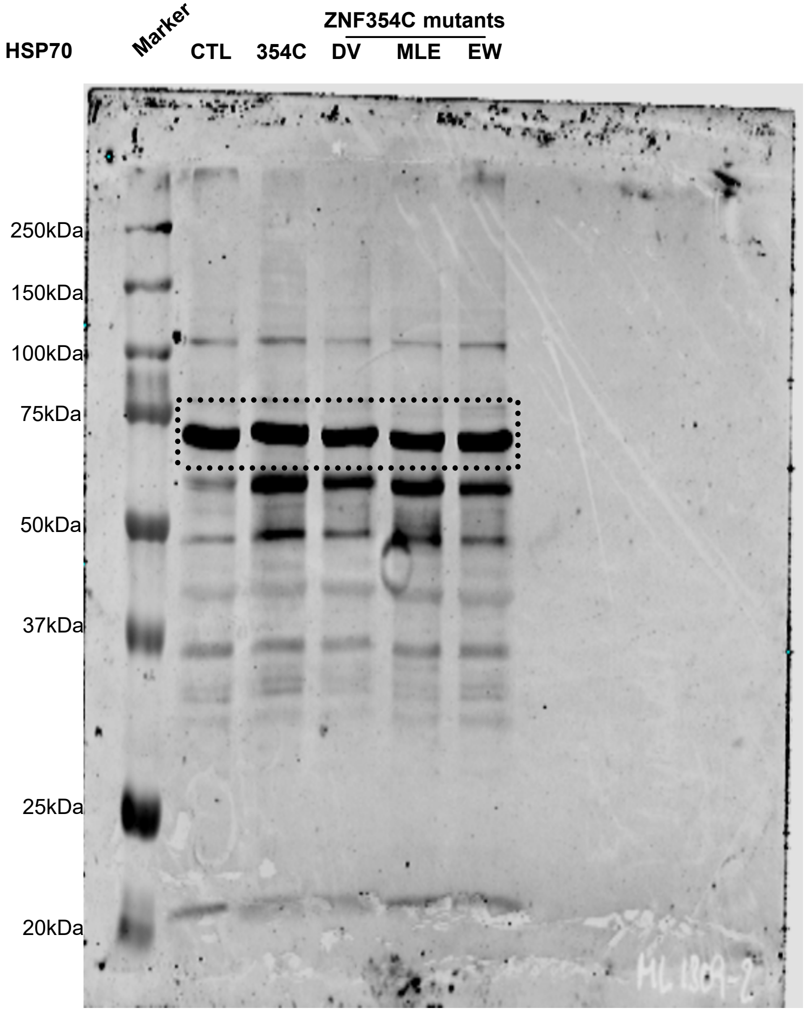
HSP70
Marker
CTL
354C
ZNF354C mutants
DV
MLE
EW
250kDa
150kDa
100kDa
75kDa
50kDa
37kDa
25kDa
20kDa

Fig. 4C

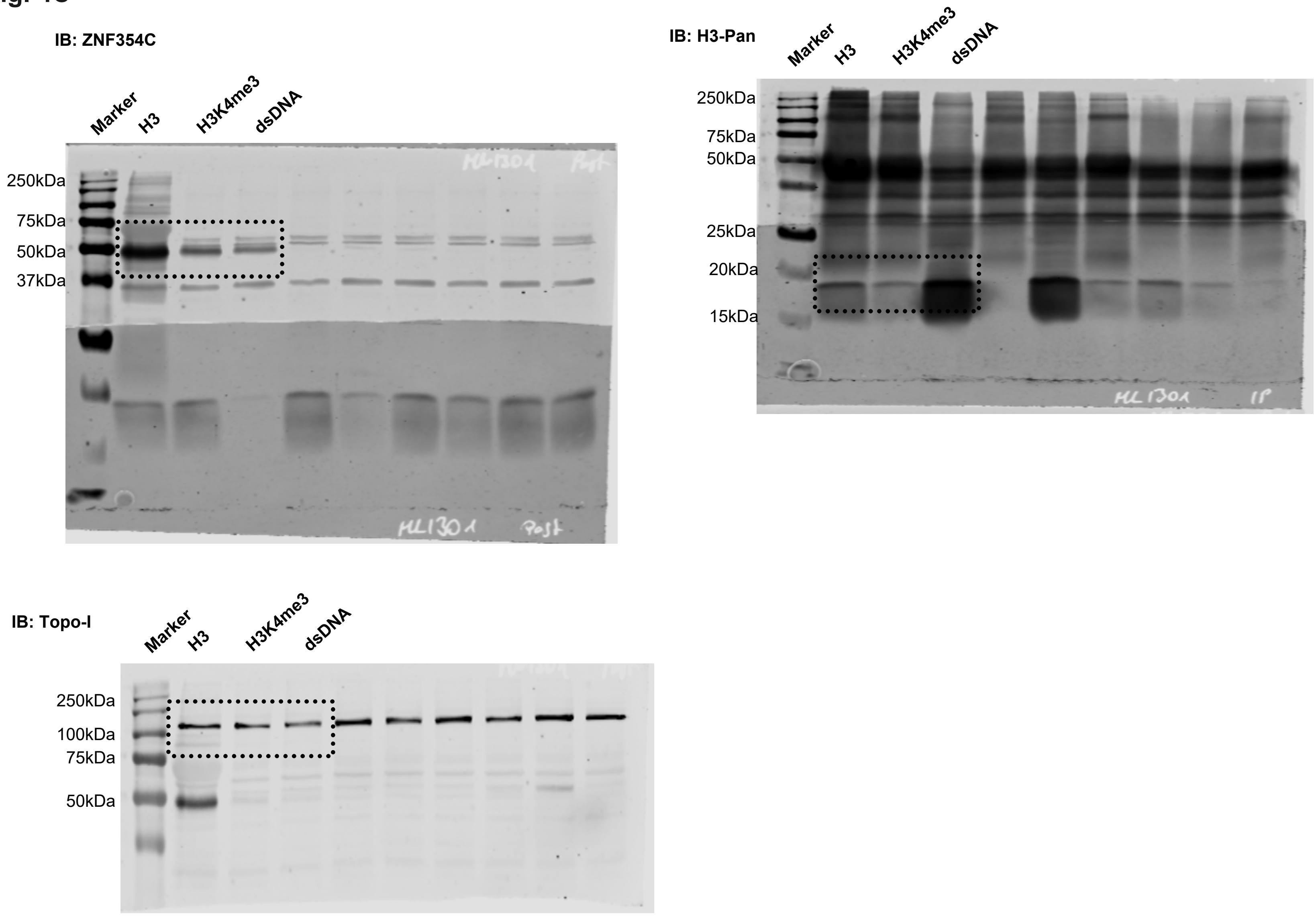
IB: ZNF354C
Marker
H3
H3K4me3
dsDNA
250kDa
75kDa
50kDa
37kDa
IB: H3-Pan
Marker
H3
H3K4me3
dsDNA
250kDa
75kDa
50kDa
25kDa
20kDa
15kDa
IB: Topo-I
Marker
H3
H3K4me3
dsDNA
250kDa
100kDa
75kDa
50kDa

Fig. 5B

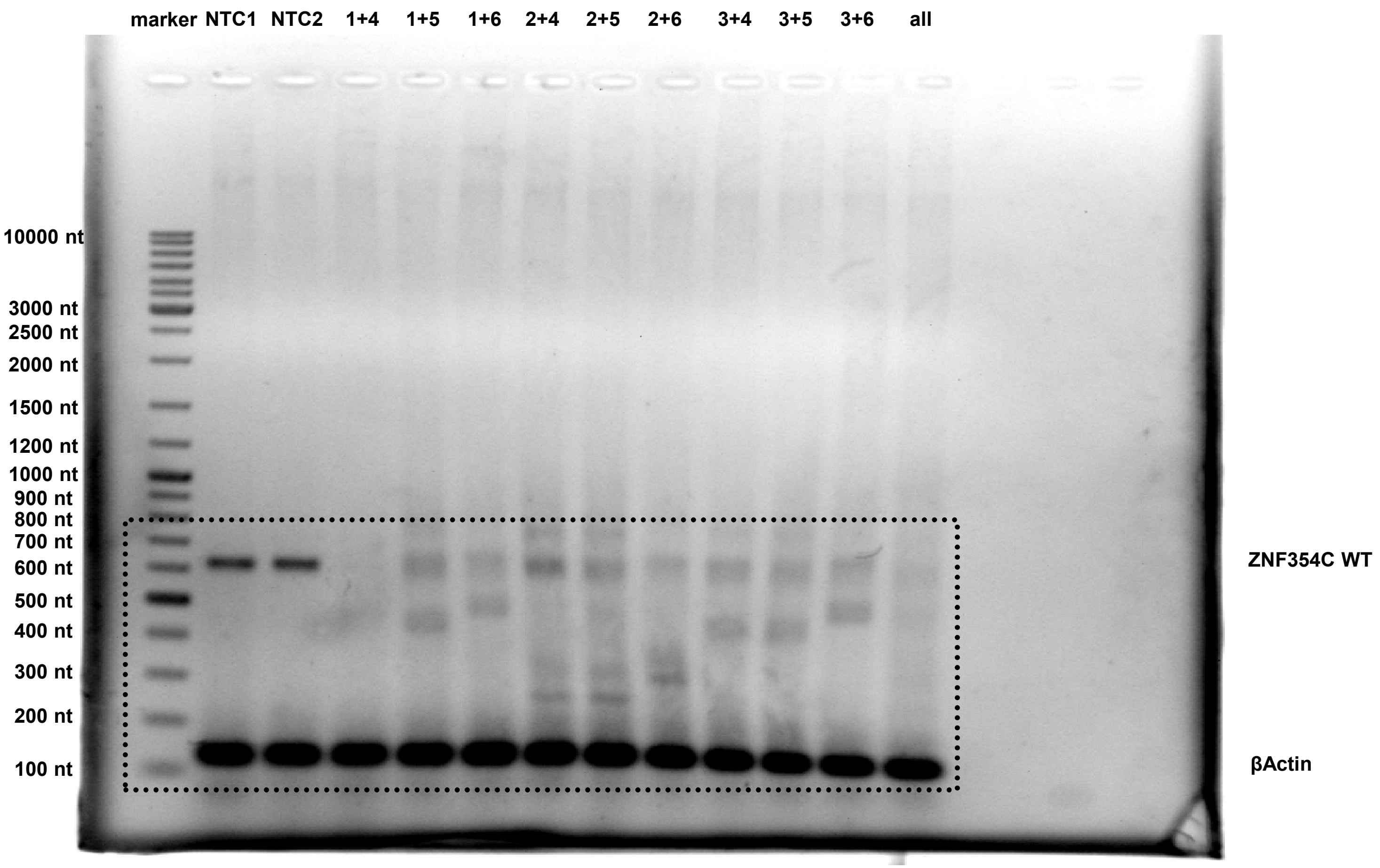
marker
NTC1
NTC2
1+4
1+5
1+6
2+4
2+5
2+6
3+4
3+5
3+6
all
10000 nt
3000 nt
2500 nt
2000 nt
1500 nt
1200 nt
1000 nt
900 nt
800 nt
700 nt
600 nt
500 nt
400 nt
300 nt
200 nt
100 nt
ZNF354C WT
βActin

Fig. 5C (Left)

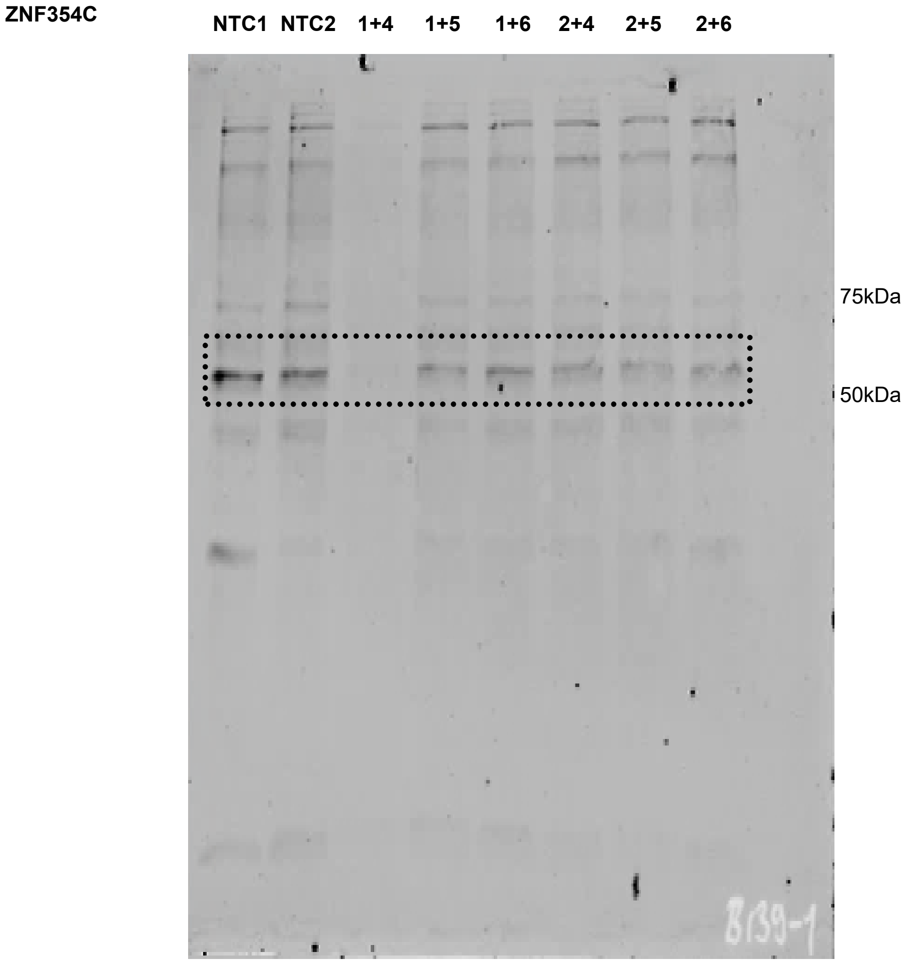
ZNF354C
NTC1
NTC2
1+4
1+5
1+6
2+4
2+5
2+6
75kDa
50kDa

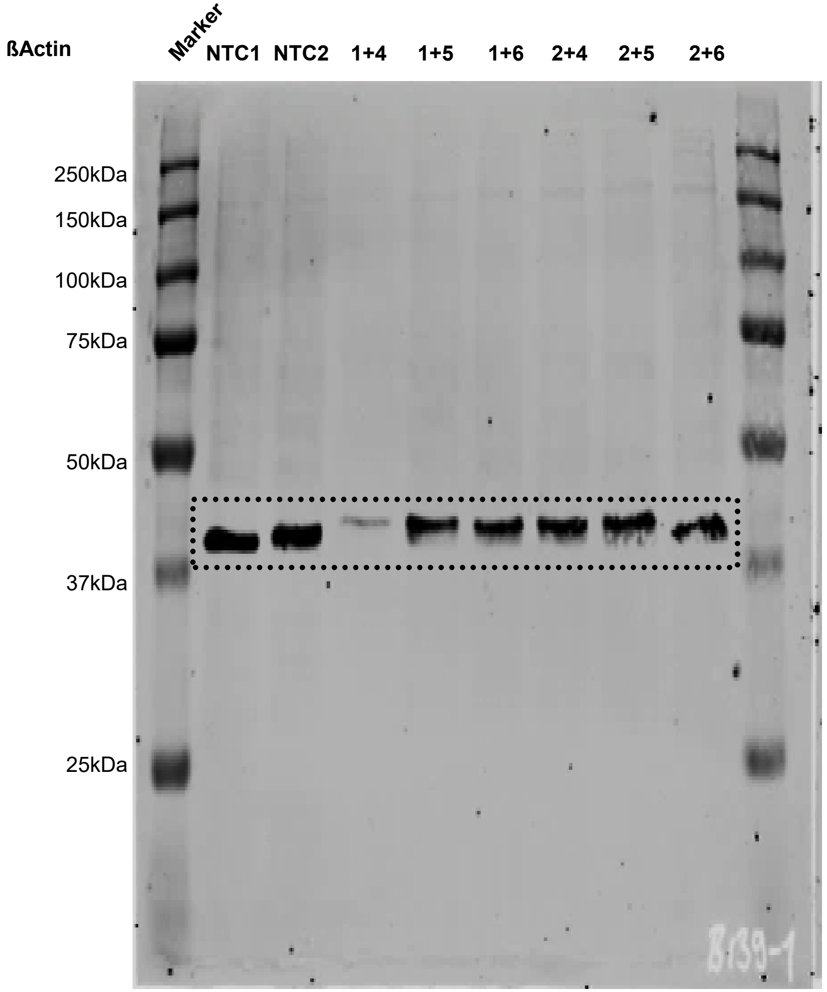
ßActin
Marker
NTC1
NTC2
1+4
1+5
1+6
2+4
2+5
2+6
250kDa
150kDa
100kDa
75kDa
50kDa
37kDa
25kDa

Fig. 5C (Right)

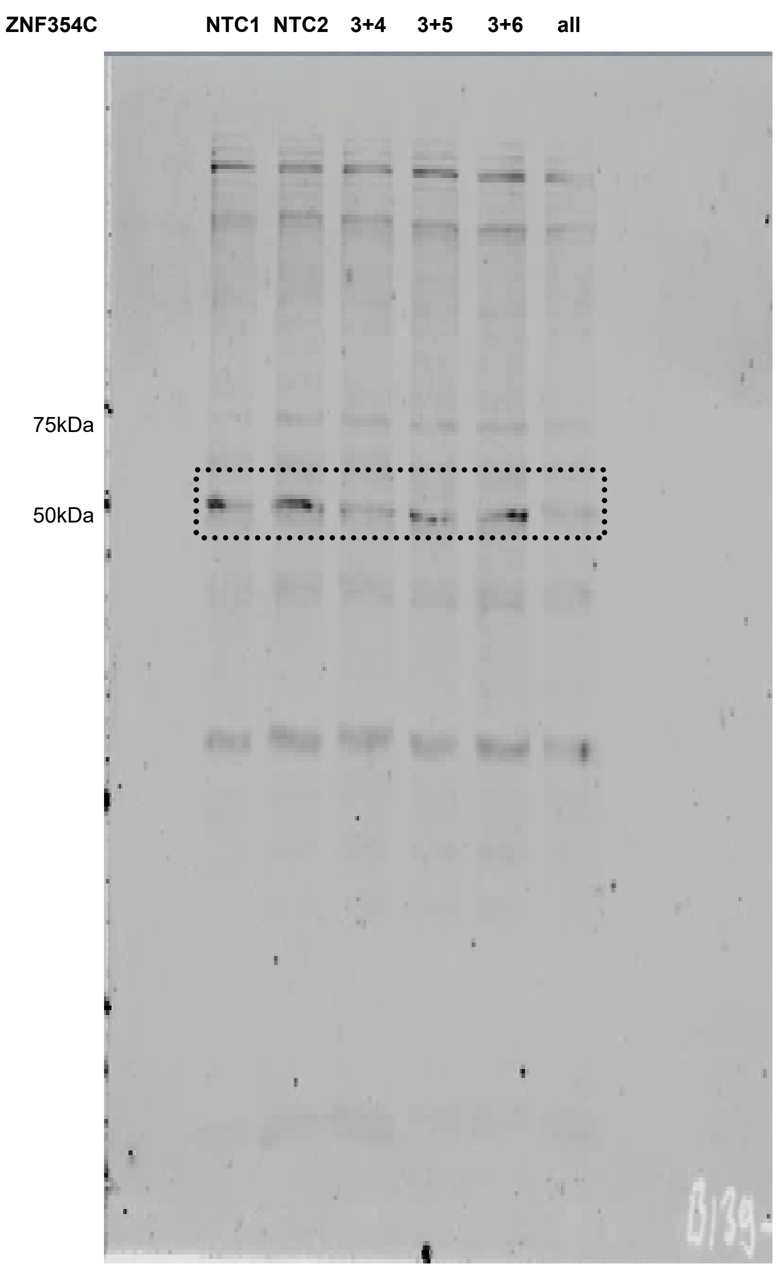
ZNF354C
NTC1 NTC2 3+4 3+5 3+6 all
75kDa
50kDa

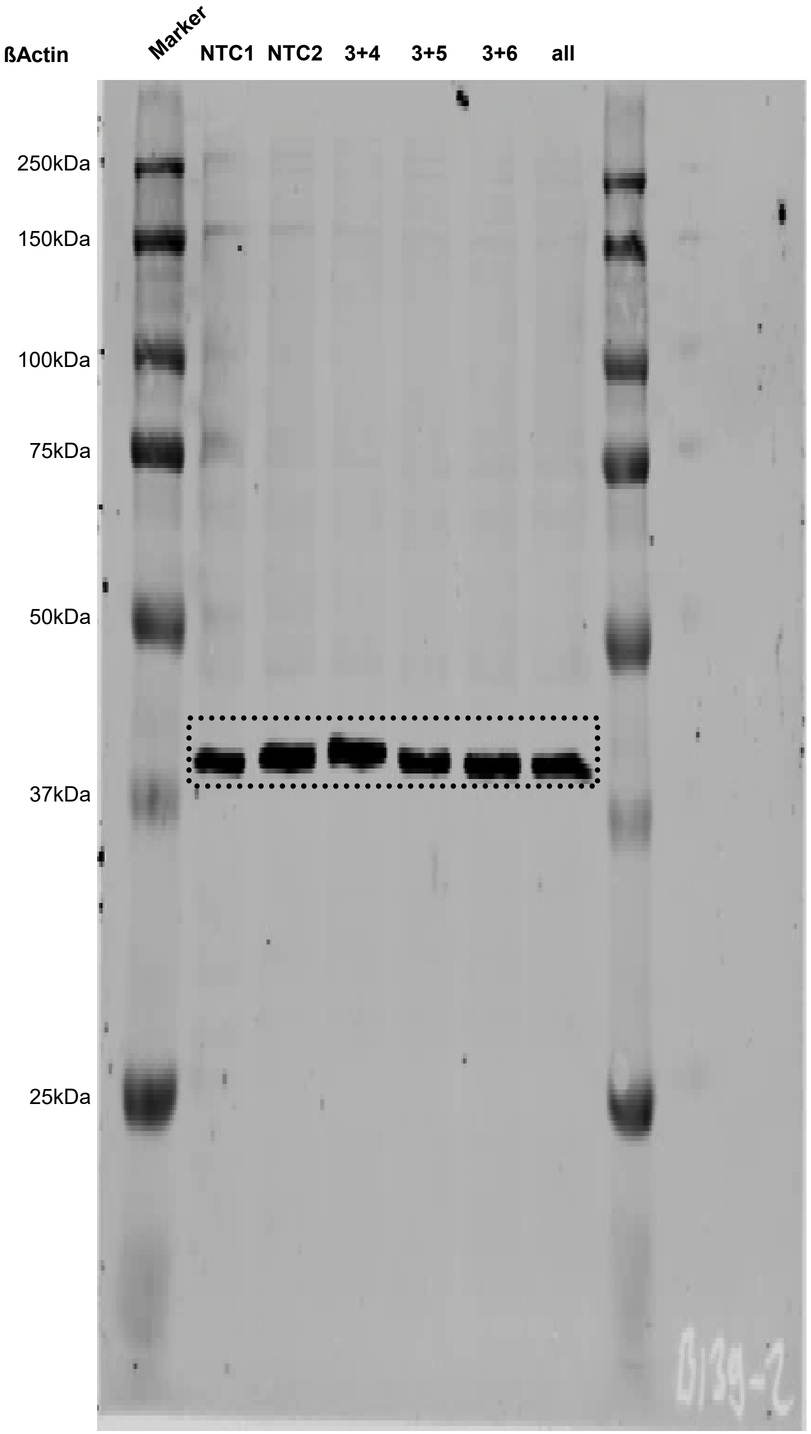
ßActin
Marker NTC1 NTC2 3+4 3+5 3+6 all
250kDa
150kDa
100kDa
75kDa
50kDa
37kDa
25kDa

Fig. 5F

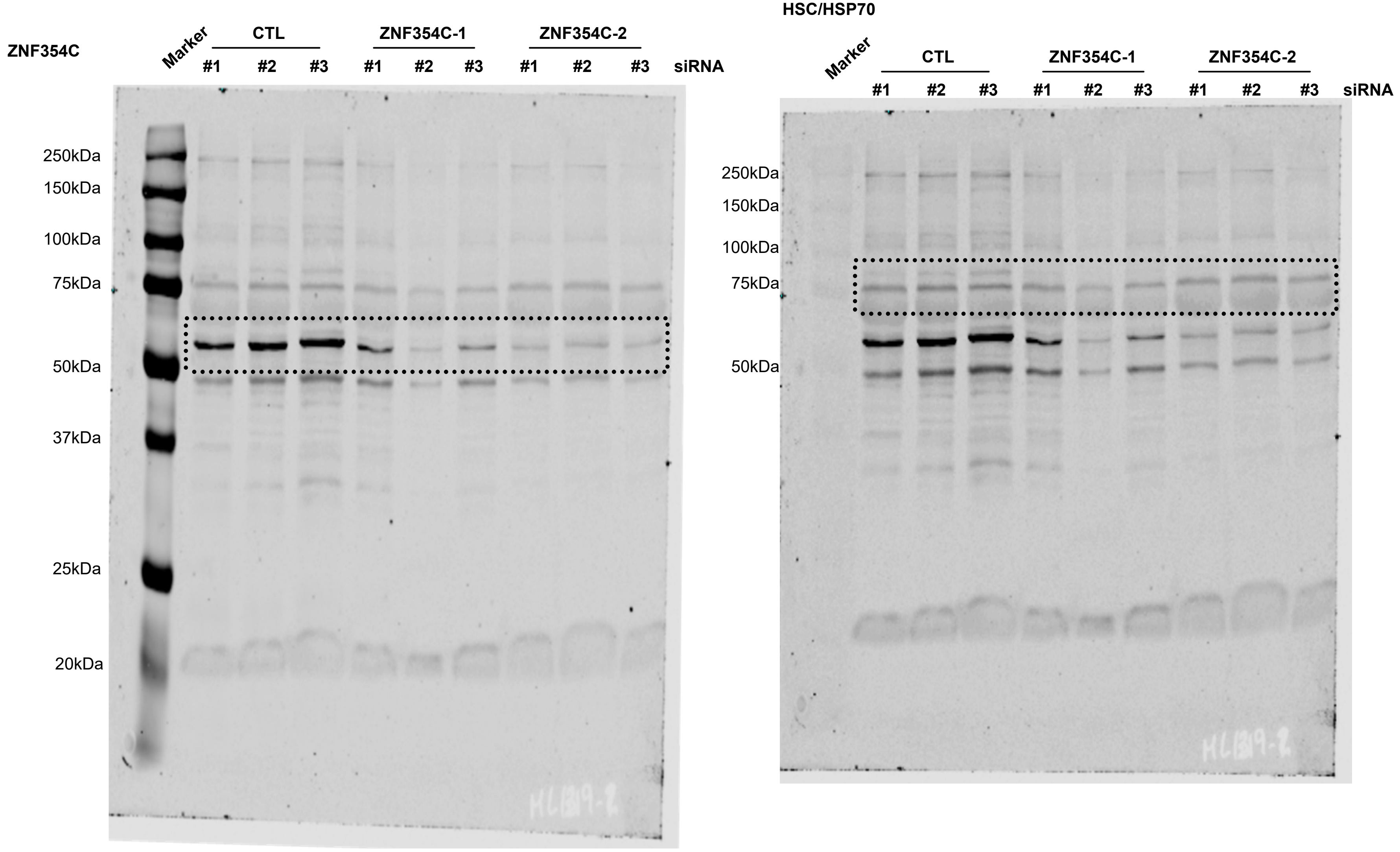

ZNF354C
Marker
CTL
ZNF354C-1
ZNF354C-2
#1 #2 #3 #1 #2 #3 #1 #2 #3 siRNA
250kDa
150kDa
100kDa
75kDa
50kDa
37kDa
25kDa
20kDa
HSC/HSP70
Marker
CTL
ZNF354C-1
ZNF354C-2
#1 #2 #3 #1 #2 #3 #1 #2 #3 siRNA
250kDa
150kDa
100kDa
75kDa
50kDa

Sup. Fig. 3B

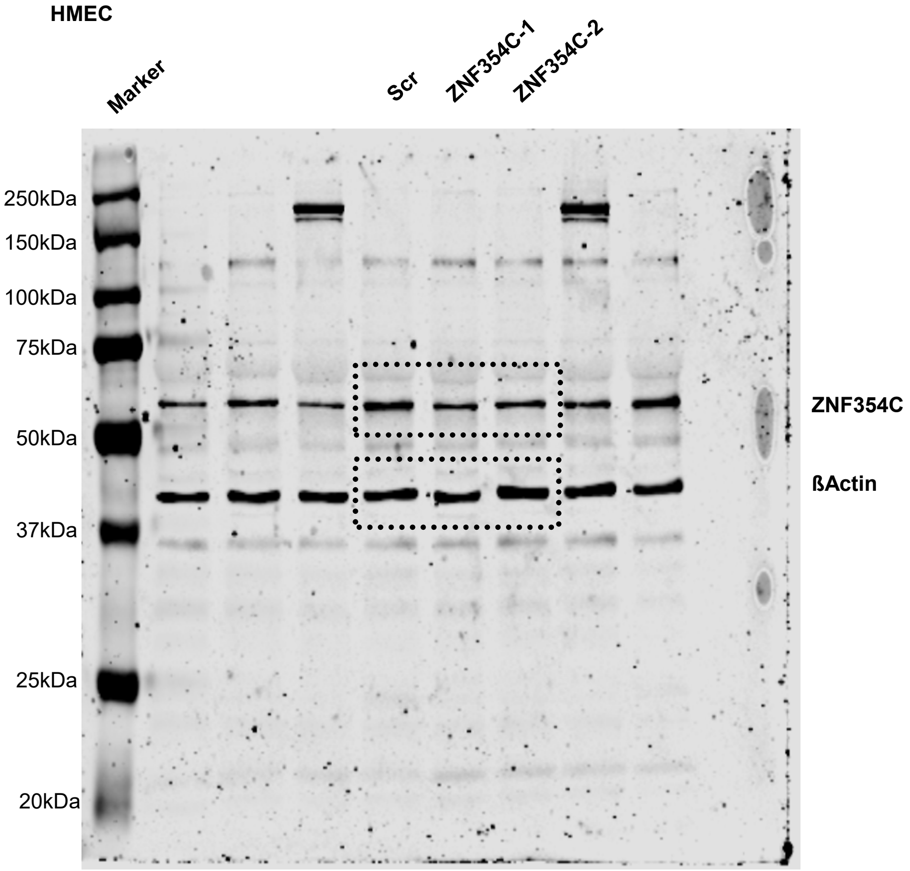
HMEC
Marker
Scr
ZNF354C-1
ZNF354C-2
250kDa
150kDa
100kDa
75kDa
50kDa
37kDa
25kDa
20kDa
ZNF354C
ßActin

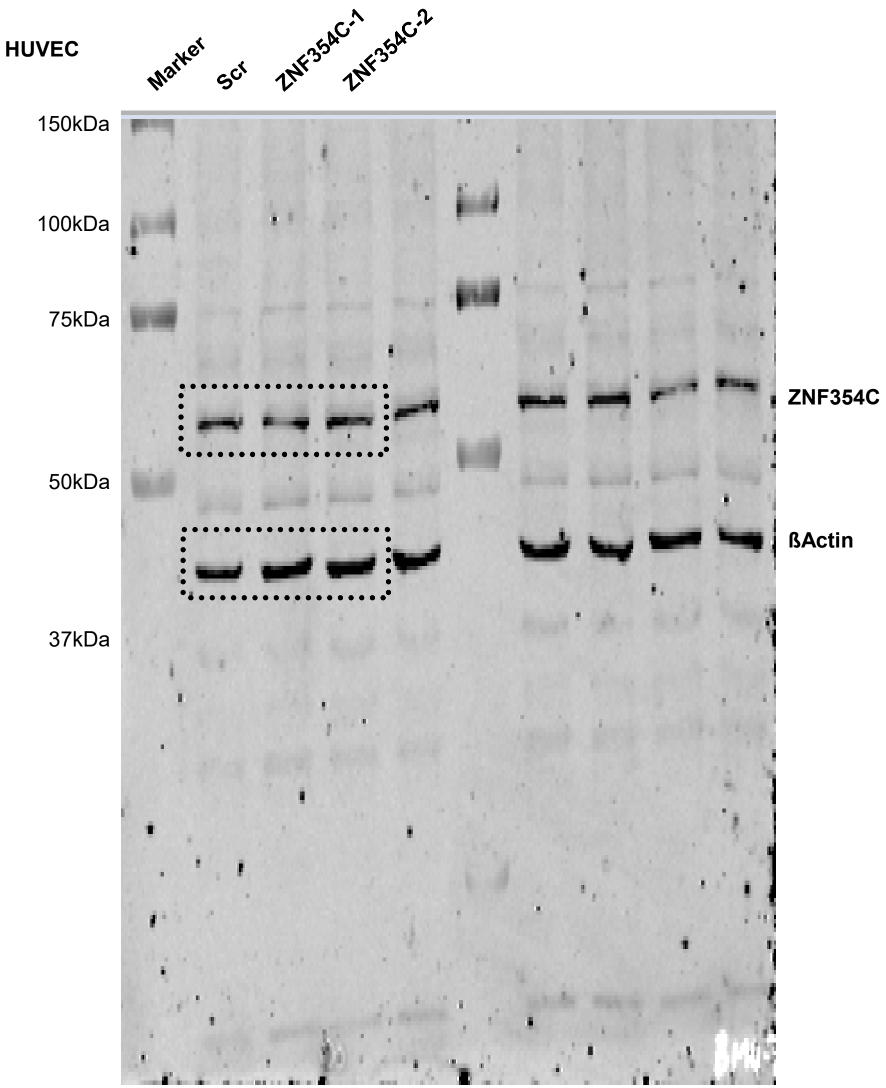
HUVEC
Marker
Scr
ZNF354C-1
ZNF354C-2
150kDa
100kDa
75kDa
50kDa
37kDa
ZNF354C
ßActin

Sup. Fig. 3B: Right HAoSMC

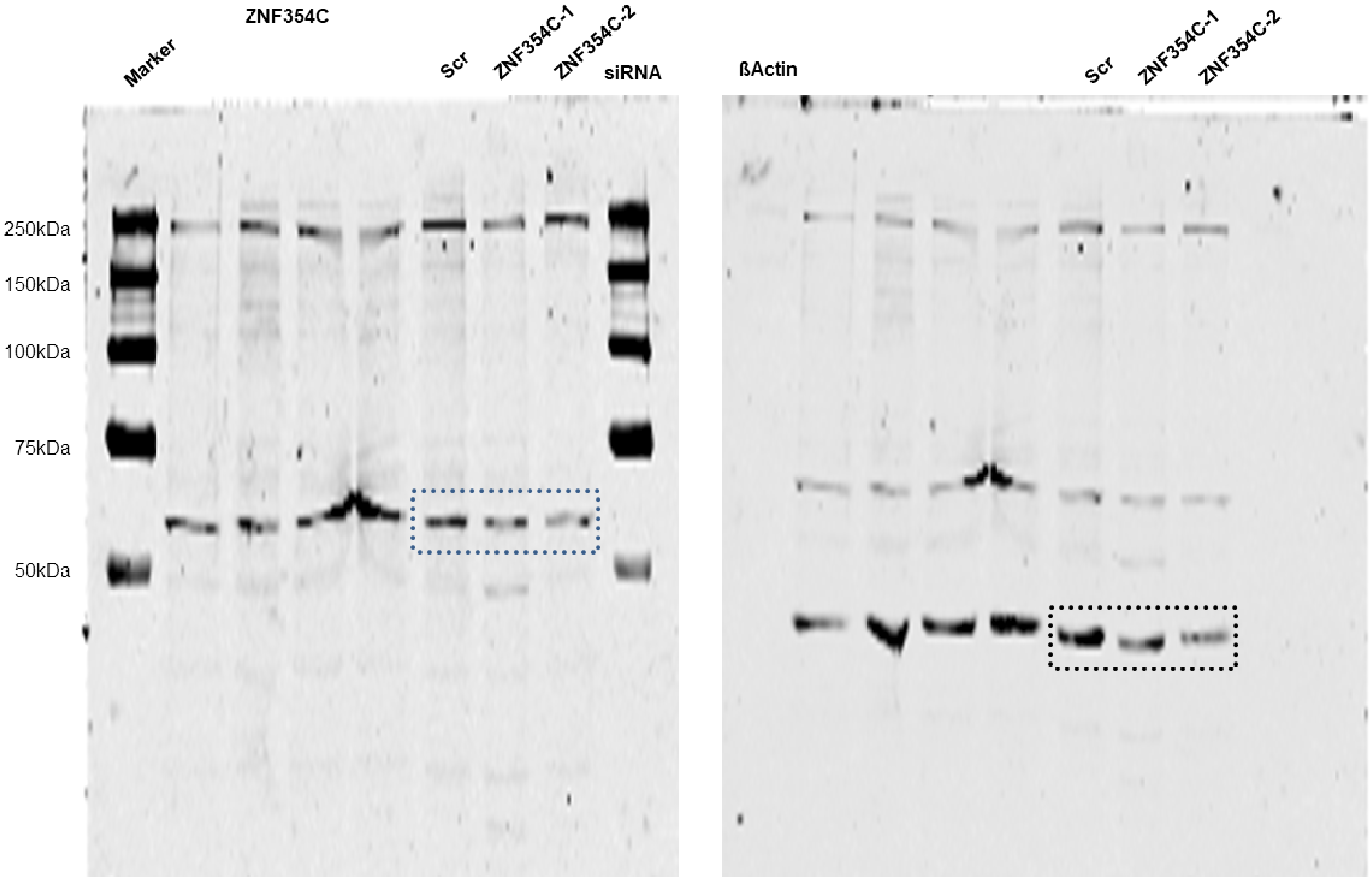

Sup. Fig. 3C

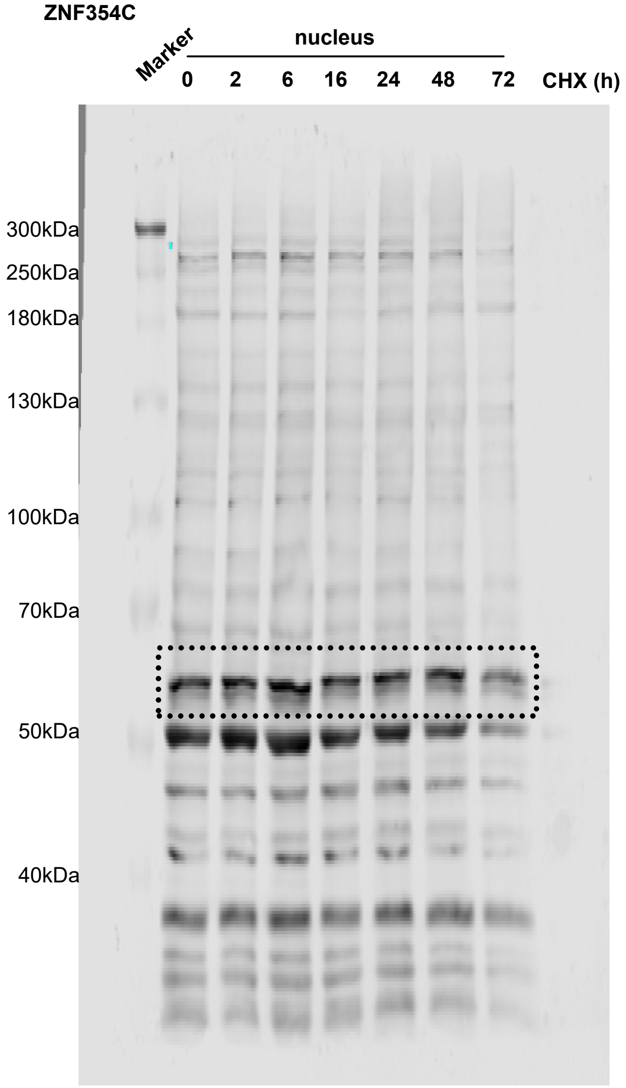
ZNF354C
Marker
nucleus
0
2
6
16
24
48
72
CHX (h)
300kDa
250kDa
180kDa
130kDa
100kDa
70kDa
50kDa
40kDa

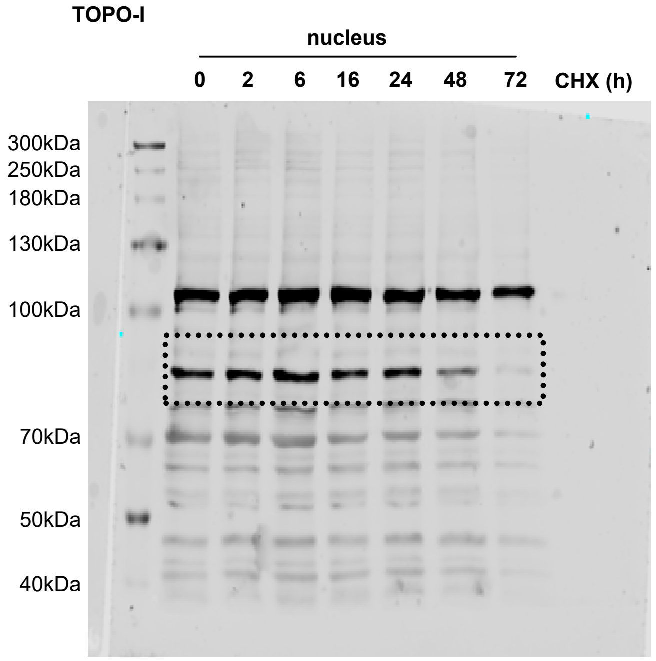
TOPO-I
nucleus
0
2
6
16
24
48
72
CHX (h)
300kDa
250kDa
180kDa
130kDa
100kDa
70kDa
50kDa
40kDa

Sup. Fig. 3D

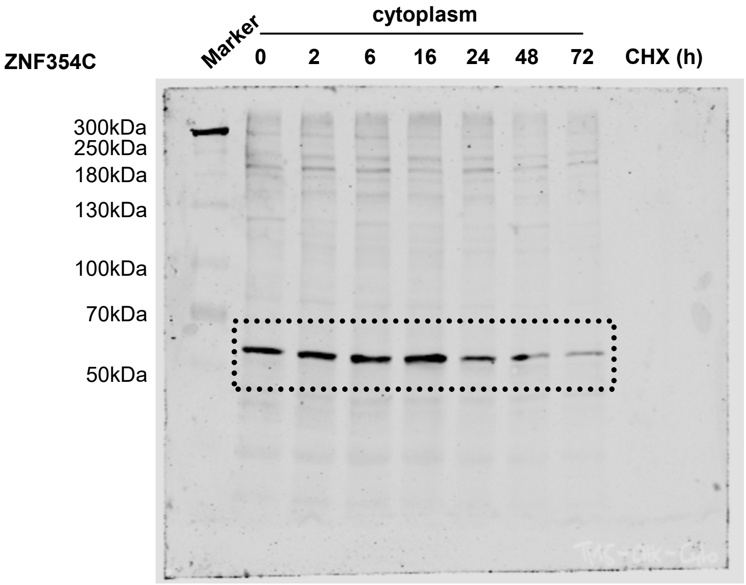
ZNF354C
Marker
cytoplasm
0
2
6
16
24
48
72
CHX (h)
300kDa
250kDa
180kDa
130kDa
100kDa
70kDa
50kDa

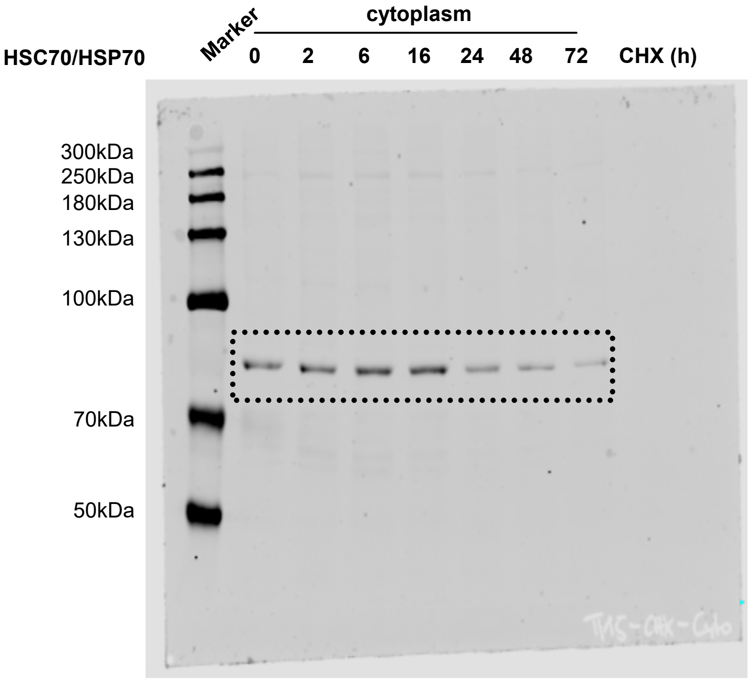
HSC70/HSP70
Marker
cytoplasm
0
2
6
16
24
48
72
CHX (h)
300kDa
250kDa
180kDa
130kDa
100kDa
70kDa
50kDa
